# White and gray matter integrity evaluated by MRI-DTI can serve as noninvasive and reliable indicators of structural and functional alterations in chronic neurotrauma

**DOI:** 10.1038/s41598-024-57706-7

**Published:** 2024-03-27

**Authors:** Lan-Wan Wang, Kuan-Hung Cho, Pi-Yu Chao, Li-Wei Kuo, Chia-Wen Chiang, Chien-Ming Chao, Mao-Tsun Lin, Ching-Ping Chang, Hung-Jung Lin, Chung-Ching Chio

**Affiliations:** 1 Department of Pediatrics, Chi Mei Medical Center, Tainan, 710 Taiwan; 2 Department of Biotechnology and Food Technology, Southern Taiwan University of Science and Technology, Tainan, 710 Taiwan; 3 Department of Electronic Engineering, National United University, Maioli City, 360 Taiwan; 4 Department of Medical Research, Chi Mei Medical Center, No. 901, Zhonghua Rd, Yongkang District, Tainan City, 710 Taiwan; 5 Institute of Biomedical Engineering and Nanomedicine, National Health Research Institutes, Miaoli County, 350 Taiwan; 6 Institute of Medical Device and Imaging, National Taiwan University College of Medicine, Taipei, 100 Taiwan; 7 Department of Intensive Care Medicine, Chi Mei Medical Center, Liouying, Tainan 73657 Taiwan; 8 Department of Dental Laboratory Technology, Min-Hwei College of Health Care Management, Tainan, 73657, Taiwan; 9 Department of Emergency Medicine, Chi Mei Medical Center, No. 901, Zhonghua Rd, Yongkang District, Tainan City, 710 Taiwan; 10 School of Medicine, Taipei Medical University, Taipei, 110 Taiwan; 11 Division of Neurosurgery, Department of Surgery, Chi Mei Medical Center, No. 901, Zhonghua Rd, Yongkang District, Tainan City, Taiwan

**Keywords:** Moderate or repetitive traumatic brain injury, White matter injury, Magnetic resonance imaging, Diffusion tensor imaging, Neurobehavioral deficits, Histopathological changes, Neurology, Medical research, Experimental models of disease, Preclinical research, Blood-brain barrier, Neuroscience, Diseases of the nervous system, Encephalopathy, Neurodegeneration, Post-traumatic stress disorder

## Abstract

We aimed to evaluate whether white and gray matter microstructure changes observed with magnetic resonance imaging (MRI)-based diffusion tensor imaging (DTI) can be used to reflect the progression of chronic brain trauma. The MRI-DTI parameters, neuropathologic changes, and behavioral performance of adult male Wistar rats that underwent moderate (2.1 atm on day “0”) or repeated mild (1.5 atm on days “0” and “2”) traumatic brain injury (TBI or rmTBI) or sham operation were evaluated at 7 days, 14 days, and 1–9 months after surgery. Neurobehavioral tests showed that TBI causes long-term motor, cognitive and neurological deficits, whereas rmTBI results in more significant deficits in these paradigms. Both histology and MRI show that rmTBI causes more significant changes in brain lesion volumes than TBI. In vivo DTI further reveals that TBI and rmTBI cause persistent microstructural changes in white matter tracts (such as the body of the corpus callosum, splenium of corpus callus, internal capsule and/or angular bundle) of both two hemispheres. Luxol fast blue measurements reveal similar myelin loss (as well as reduction in white matter thickness) in ipsilateral and contralateral hemispheres as observed by DTI analysis in injured rats. These data indicate that the disintegration of microstructural changes in white and gray matter parameters analyzed by MRI-DTI can serve as noninvasive and reliable markers of structural and functional level alterations in chronic TBI.

## Introduction

Based on increasing clinical and experimental evidence, traumatic brain injury (TBI) is correlated with progressive histological damage in research on humans and animal models of TBI are more suited for studying the mechanisms of chronic TBI. In 2020, Leconte and colleagues^1^ assessed the behavioral and histopathological effects of a single cortical contusion up to 10 months after TBI in mice. Their study evaluated motor and memory functions and depression-like behavior concomitantly, and magnetic resonance imaging (MRI) was repeatedly performed. They suggested that experimental TBI models in mice mimic the long-term sequelae of human brain injury, such as neurological and neuropsychiatric disorders^1^.

MRI has been increasingly used clinically to better understand the secondary damage following TBI^2–4^. Diffusion tensor imaging (DTI) measures the magnitude and directionality of water diffusion, evaluates tissue microstructure, and provides a more accurate evaluation of secondary damage^5–7^. Although Laitinen and colleagues^8^ revealed persistent microstructural tissue changes in white matter tracts using mainly ex vivo DTI in a rat model of TBI, it still needs more preclinical in vivo data to ascertain whether white and gray matter integrity evaluated by MRI-DTI can serve as noninvasive and reliable indicators of structural and functional alternations in chronic neurotrauma. Since 1994, at least 145 publications have utilized DTI to detect TBI-related pathology^9^; however, again needs to fill the gap relating behavioral outcomes with pathologic outcomes and also DTI outcomes in several brain regions in chronic TBI. Furthermore, clinical evidence shows repeated mild TBI increases morbidity risk and long-term neurodegenerative pathology such as chronic traumatic encephalopathy^10,11^. Repeated mild TBI (rmTBI) also induced atrophy of the corpus callosum in the chronic post-injury period. We aimed to specifically implicate repeated injury as a causative factor. So, in this study, animal models of a single moderate TBI were directly compared with repeated mild TBI within the same study.

The objective of this study was to describe and compare the neurobehavioral and pathological consequences in a rat model of single moderate (2.0–2.1 atm) TBI or repetitive mild injury (rmTBI, two injuries given at 48 h intervals) administered by lateral fluid percussion (LFP) to ascertain whether a correlation exists between the white and gray matter loss and secondary brain damage. In particular, neurobehavior, MRI-DTI, and histologic analyses were performed on the same animals to evaluate the damage to both hemispheres of brain at the following time points: 1 day before the operation and 7 days (D7), 14 days (D14), 1 month (M1), 3 months (M3), 6 months (M6), and 9 months (M9) following surgery. Neurobehavioral performance was evaluated during the whole process. The blood–brain barrier (BBB) and brain microstructures were evaluated by contrast-enhanced T1-weighted imaging (CE-T1WI)^12^ and MRI-DTI^13^, respectively. Luxol Fast Blue (LFB) and cresyl violet staining were used to identify myelin and neuronal cell bodies, respectively^14^. We further determined the amounts of ankyrin G and contactin-associated proteins (Caspr) at the nodes of Ranvier^15^.

## Methods

### Animal studies and ethics statement

Adult male, specific pathogen-free Wistar rats (aged 10 weeks and weighing 380–420 g) were obtained from the BioLASCO Taiwan Co., Ltd. (Taipei, Taiwan) and housed at the Central Animal Facility of the Chi Mei Medical Center. Only male rats were used in these studies, as they were not designed to analyze sex differences. The animals were housed in groups of four at an ambient temperature of 22 ± 1 °C, with a 12 h light–dark cycle. Rat chow and tap water were available ad libitum. The Institutional Animal Care and Use Committee at Chi Mei Medical Center approved all experimental procedures (IACUC Approved Number: 105122608). All animal experiments were designed in accordance with the ARRIVE guidelines. Three hundred forty-five male Wistar rats were divided into sham, TBI, and rmTBI groups using randomization methods (https://www.randomizer.org/). Rats were identified by a number printed (use a nontoxic, temporary marker pen) on the base of the tail before surgery and used by Stoelting™ Rat Ear Tag (Stoelting Co., Il, USA) for identification post-surgery.

### Rat model of TBI

Rats were surgically prepared for LFP following the methodology detailed previously^16–18^. Rats were randomly divided into three groups: sham, TBI, and rmTBI. The TBI group rats received single moderate (2.0–2.1 atm) LFP on day 0 (D0), while the rmTBI group rats received repeated mild (1.5 atm) LFP on days 0 and 3 (D0 and D3). All animals were intraperitoneally injected with Zoletil^®^ 100 (20 mg/kg; Virbac, Carros, France) and a mixture containing balancing (1%, Health-Tech Pharmaceutical Co., Ltd), and atropine sulfate (0.02 mg/kg, Tai Yu Chemical & Pharmaceutical Co., Ltd, Taiwan) before surgery. The rat was placed in a stereotaxic frame, and the scalp was incised sagittally. After an incision was made in the scalp, a 4.8 mm circular craniotomy was performed at A/P − 3 mm, and M/L 4 mm with reference to bregma^19^, and 3.0 mm to the right of the central suture over the right parietal cortex. A modified leur-lock connector (trauma cannula), 2.6 mm inner diameter, was cemented into the craniotomy with dental acrylic and then connected to a fluid percussion device (VCU Biomedical Engineering, Richmond, VA, USA). Sham animals were connected to the LFP device, but no fluid percussion was delivered. Postimpact seizures were observed immediately after the induction of LFP in ∼30% of rats and lasted for 10–15 s. Other aspects of TBI were reproduced by LFP, such as hematoma that occurred at the gray–white matter interface, acute apnea (typically 10–60 s), intracranial hypertension, bradycardia, hyperglycemia, and suppressed electroencephalogram amplitude^20^. The rmTBI group of rats was again anesthetized and received either sham or mild LFP on D3. After surgery, the cannula was removed, and the incision was sutured closed. A heating pad was used during surgery and recovery to maintain the rats' body temperature at 36.5 °C. Rats were returned to their home cages at an ambient temperature of 26 °C and appropriately fed and hydrated. Ketoprofen (5 mg/kg/BID, subcutaneous injection) was used for postoperative analgesia. Figure 2A showed that at the end of M9 (or 270 days), compared to the sham group rats (100% survival rate), the TBI group and the rmTBI group rats had 40% survival and 80% survival rate, respectively (Fig. 2A).

For long-term consequences observation, rat brain samples were obtained at 7 and 14 days and 1, 3, 6, and 9 months after sham, TBI, and rmTBI treatments.

### Study design

**Figure 1:**
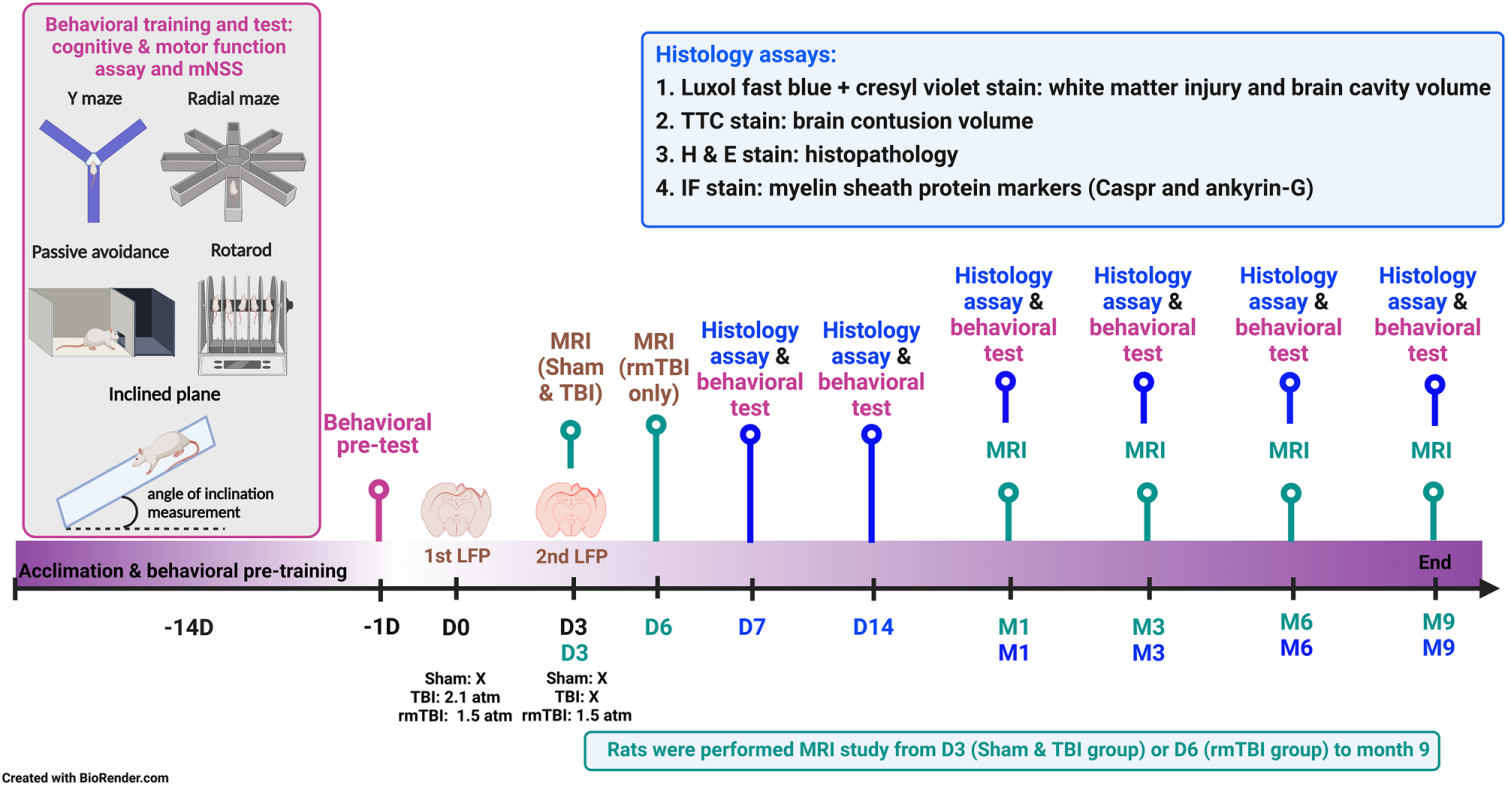
Outline of the experimental schedule. Wister rats were randomized into sham operation, moderate TBI (TBI), and repetitive mild TBI (rmTBI) groups. Fourteen days before surgery, rats were acclimated to all behavior testing environments and pretraining, including the Y maze, radial maze, passive avoidance, rotarod, inclined plane, and modified neurological severity score (mNSS). One day before surgery, the neurobehavioral pretests were performed. On day 0 (D0), rats received a sham operation or a single TBI 2.1 atm) or rmTBI (1.5 atm). Three days later (D3), the rmTBI group received another single rmTBI (1.5 atm). The MRI observation was performed from D3 (sham and TBI groups) or D6 (rmTBI group) to 9 months (M9). The behavioral tests were performed from D7 to M9. Then, the rats were sacrificed and the brains were removed for histological assays.

All rats were allowed to acclimate to the accredited facility for at least 7 days before beginning any behavioral procedures. For one week prior to the testing day, the rats were placed on the Y maze, radial maze, rotarod, and passive avoidance apparatus for 3 days to explore the environment for 10 min daily and then trained for 3 days. One day before surgery (-1D), rats received behavioral pretesting. At the indicated times following the surgery, the animals were subjected to MRI study from day 3 (sham and TBI groups) or day 6 (rmTBI group) to month 9. The behavioral tests and histological analysis were performed from day 7, day 14, month 1 to month 9 following the first impact or surgery to ensure the direct comparison between MRI images, neurobehavioral performance, and histological data (Fig. 1). Behavioral, MRI, and histological assessments were performed by researchers who were blinded to the experimental groups.

The animals that suddenly died immediately after the surgery (< 5%) were excluded from the study. The average survival rate up to 9 months was approximately 73% (100% in the sham group, 40% in the TBI group, and 80% in the rmTBI group). Animals were monitored daily to ensure survival, intake and output conditions, and body weight. We observed pathophysiological changes in the brain microenvironment at the acute (3–7 days) and chronic (6–9 months) phases following TBI using MRI, neurobehavioral tests, and immunohistochemistry methods.

### Neurobehavioral function

#### Passive avoidance assay^21^

The training apparatus had two compartments comprising a small chamber (25 × 25 × 20 cm) and a large dark compartment (50 × 25 × 20 cm). A guillotine door separated these two compartments. Electric shocks were delivered to the grid floor by an isolated stimulator. At the beginning of the test, each rat was placed in the apparatus for 5 min to become habituated. An acquisition trial was performed; rats were placed individually in the illuminated chamber. After a habituation period (1 min), the guillotine door was lifted. Then after the rat entered the dark chamber, the door was lowered across the dark compartment, and preshock latency was recorded. After exposure to the foot shock, the rat was moved from the passive avoidance apparatus to its home cage. Retention of passive avoidance performance was tested 24 h afterward. The rat was placed in the lighted (safe) compartment again with access to the dark compartment without any shock. The latency to enter the dark compartment was measured (i.e., testing latency) up to a maximum of 300 s.

#### Radial maze assay^22^

The maze comprised of 8 arms, extending radially from a central area. Before the training, the rats were allowed to explore the maze for 5 min and freely consume food. The animals were trained for five days to run to the end of the arms and consume the baited food. The training trial continued until 5 min had passed. After adaptation, each animal was checked for working memory errors (entrance into an arm containing food, but previously entered) and reference memory errors (entrance into an arm that was never baited), in which the same four arms (no. 2, 4, 6, and 8) were baited. All the rats were trained with one trial per day for five consecutive days before surgery and one trial per week for five consecutive weeks after surgery. Finally, the number of long-term memory (reference memory) errors and short-term memory (working memory) errors were counted^22^.

#### Y maze assay

Spontaneous alternation behavior requiring attention and working memory in a Y-maze was assessed by the methods of Sarter et al.^23^. Each arm of the Y-maze was 45 cm long, 10 cm wide, and 35 cm high, and all arms were positioned at equal angles. Each rat was placed at the end of an arm and allowed to enter the maze freely for an 8-min test session without reinforcers such as food, water, or electric shock. An arm entry was defined as the entry of all four paws into one arm. The sequence of arm entries was recorded with a video camera. The alternation behavior (actual alternations) was defined as the consecutive entry into three arms, i.e., the combination of three different arms, with stepwise combinations in the sequence. The maximum number of alternations is thus the total number of arms entered minus 2, and the percentage of alternation behavior was calculated as (alternations/ maximum alternations) × 100.

#### Rotarod assay^24^

A rotarod treadmill (ENV576; Med Associates, St Albans, VT, USA) was used to test the motor coordination of the rats prior to surgery and in the postsurgical recuperation phase. The rotarod was set to accelerate over a 5 min period from 4 to 30 rpm; the maximal time performance of each rat was recorded (maximum: 5 min). The value reported for each animal is the percentage of the baseline values.

#### Modified neurological severity score (mNSS)^25^ and incline plane^26^

mNSS and incline plane tests were assessed before and during the whole experimental period. The mNSS is a composite of motor (muscle status, abnormal movement), sensory (visual, tactile, and proprioceptive), and reflex tests. One point was given for failure to perform a task. Thus, the higher the score, the more severe the injury, with a maximum of 18 points. Briefly, mNSS score can evaluate neurological damage after TBI, including motor, sensory, reflex, and balance function of the experimental animals, and monitor the functional recovery after TBI. The score ranges from 0 to 18, and the severity can be defined as 13–18, severe injury; 7–12, moderate injury; 1–6, mid injury.

### MRI

The MRI experiments in this study were conducted using a custom-integrated 3 T MRI system, included a high-strength gradient coil with a maximum strength of 675 mT/m and a maximum slew rate of 6750 mT/m/ms (BFG 200/115 S14, Resonance Research Inc., Billerica, MA, USA). This gradient coil was integrated with the magnet from a previously used clinical 3 T MRI scanner (Achieva, Philips Healthcare, Best, Netherlands) to enable high-resolution imaging of small animals.The control of the hardware, imaging sequences, and MRI parameter settings was managed by a commercial spectrometer (EVO, MR Solutions Ltd., Guildford, Surrey, UK) in conjunction with bundled console software (PowerScan, MR Solutions Ltd., Guildford, Surrey, UK). For the acquisition of MR signals, a single-loop transmit/receive surface coil with a diameter of 35 mm (Doty Scientific, Columbia, SC, USA) was utilized. Further information regarding the integration, calibration, and validation of this 3 T MRI system can be found in our previous publication^27^.

T2 weighted images (T2WI) were acquired using a fast spinecho sequence with the following parameters: repetition time (TR) = 4000 ms, echo time (TE) = 68 ms, 20 slices with a thickness of 1 mm, field of view (FOV) = 40 × 40 mm, matrix size = 256 × 256 (in-plane resolution of 156 × 156 μm), and 8 averages. A spinecho sequence was used for precontrast T1 weighted images (T1WIs) with TR = 640 ms, TE = 11 ms, 16 slices with a thickness of 1 mm, FOV = 40 × 40 mm, matrix = 192 × 192 (in-plane resolution of 208 um) and 4 averages. The postcontrast T1WI was acquired using the same parameters as precontrast T1WI imaging immediately after intravenous injection of gadobutrol 1.0 (GADOVIST™, 1 mmol/mL, Bayer Pharmaceuticals, USA) in a bolus dose of 0.1 mmol/kg through a cannula. The percentage of signal change is defined as follows:

$$\begin{aligned} & {\text{Percentage}}\;{\text{of}}\;{\text{signal}}\;{\text{change}} \\ & \quad = \left( {{\text{post}} - {\text{contrast}}\;{\text{signal}} - {\text{pre - contrast}}\;{\text{signal}}} \right)/{\text{pre - contrast}}\;{\text{signal}} \times {1}00\% . \\ \end{aligned}$$

We employed the pulse gradient spin echo (PGSE) sequence for acquiring DTI data, specifically to mitigate common EPI (echo-planar imaging) artifacts, with TR = 2000 ms, TE = 27 ms, same slices as used in T2WI, and matrix size = 128 × 128 (in-plane resolution of 312 × 312 um). These artifacts encompass susceptibility artifacts, eddy current effects, b0 inhomogeneity-induced geometric distortions and signal loss. Consequently, there was no need for post-processing procedures to correct EPI artifacts in our DTI data. Additionally, we refrained from applying motion correction to our DTI data, as the rat head was meticulously secured during image acquisition, and no discernible movement was observed. As a result, no post-processing was undertaken for our DTI dataset. The diffusion time, diffusion gradient pulse duration, and gradient strength were set to 15 ms, 3.5 ms, and 29.1 G/cm, respectively, yielding a b value of 1000 s/mm^2^. Five b0 images and 20 noncollinear diffusion directions were encoded, and the total scan time was approximately 2 h. The diffusion tensor and its four quantitative indices, axial diffusivity (AD), radial diffusivity (RD), mean diffusivity (MD), and fractional anisotropy (FA)^28^ were reconstructed from the acquired data by using an in-house program written in C++.

The regions of interest (ROIs) were manually outlined based on rat brain atlas maps^29,30^ by two of the authors (Dr. Li-Wei Kuo and Dr. Kuan-Hung Cho) with extensive experience in neuroanatomy, both in histologic preparation and MRI. They have developed a proprietary code that has been successfully employed for DTI reconstruction in previous publications^27,31^. In this study, the white matter regions were selected by reference to the paper of Laitinen et al.^8^ and were manually outlined on FA maps (Supplementary Fig. S1). The gray regions were outlined on T2WI (Supplementary Fig. S2) by reference to the rat brain atlas^30^. The selected ROIs included the gray matter (e.g., cortex, hippocampus, and striatum) and white matter (e.g., angular bundle [ab], body of the corpus callosum [CCb], splenium of the corpus callosum [CCs], and internal capsule [ic]). After M9 MRI, rats were sacrificed, and the brain were removed for triphenyl tetrazolium chloride (TTC) staining.

### Cerebral contusion volume analyses

After the injury, all the animals were euthanized with a Zotil overdose and then transcardially perfused with 200 ml 0.9% NaCl containing1 U/ml heparin (Sigma-Aldrich) followed by 200 ml of 10% neutral buffered formalin (Sigma-Aldrich). Brains were removed, postfixed in formalin (Sigma-Aldrich), processed, and embedded in paraffin. Coronal Sects. (1 mm thick) were cut and stained with TTC (Alfa Aesar, Ward Hill, MA, USA) for light microscopic analyses of brain lesions. The contusion volume, as revealed by negative TTC stains indicating dehydrogenase-deficient tissue, was measured in each slice and summed using computerized planimetry (Image ProPlus, Media Cybernetics, MD USA. RRID: SCR_007369) and calculated as 1 mm (thickness of the slice) × (sum of the contusion area in all brain slices: mm^2^)^32^. For volumetric assessment of this brain lesion, digitized images of brain sections, taken every millimeter from 1 to 15 mm from anterior to posterior of the brain, were captured. All imaging and contusion volume analyses were performed by an independent investigator blinded to postinjury treatment status.

### Brain tissue sectioning methodology for hematoxylin and eosin (HE), LFB, and immunofluorescence (IF) staining analysis in sham, TBI, and rmTBI rats

Ten rats per time point per group were used in the histological analysis. Rats were anesthetized and transcardially perfused with 4% paraformaldehyde (PFA), and the brains were removed and fixed in formalin overnight. The formalin-fixed brains were embedded in paraffin blocks. Rat brains from each group were serially sectioned coronally at a thickness of 10 μm using a paraffin microtome. From bregma + 2.2 mm to − 7.0 mm, the brain sections could be divided into five regions (#1–#5), which enabled observation of alterations in the entire cerebral cortex, hippocampus, striatum, hypothalamus, and white matter.

For region #1, nine consecutive coronal sections numbered 1, 2, 3, 4, 5, 6, 7, 8, and 9 were obtained starting from bregma + 2.2 mm and onward. Brain sections no. 1, 2, and 3 were placed on three different slides labeled HE. Nos. 4, 5, and 6 were placed on three different slides labeled LFB, and Nos. 7, 8, and 9 were placed on three different slides labeled IF. Subsequently, the next 6 consecutive slices were discarded. The other brain sections that underwent the same procedure were preserved as needed. For regions #2, #3, #4, and #5, the same procedures were repeated, and 9 sections of each region were obtained and placed on the same slides of region #1 of nine different slides labeled HE, LFB, and IF. In each brain, three slides were prepared for each stain, and regions #1 to #5 were contained in each slide. Sections were subjected to various histochemical staining procedures to observe the pathological changes (HE stain), white matter width (LFB stain), and nodes of Ranvier protein intensity (IF stain). Three brain sections per region of interest (ROI) per rat were averaged, and ten rats per group were used for quantification.

### HE staining

Brain sections were stained with HE and examined by a light microscope (Carl Zeiss Microscopy GmbH, Jena, Germany) for histological examination. A digital camera linked to a computer running Axioscope version 4 (Carl Zeiss) was used to capture images. The damage scores were determined by grading the lesion field and morphological changes. In the grading of the lesion field^33^, damage scores from 0 to 4 denote no pathological changes, score 0; lesions involving 25% of the field, score 1; lesions involving 25–50% of the field, score 2; lesions involving 50–75% of the field, score 3; and lesions involving 75–100% of the field, score 4. In the determination of the morphological change^34^, scores are from 0 to 3, which denote normal morphology (score 0); minor edema and few pyknotic cells (score 1); moderate edema, pyknotic cells, vacuolization, and inflammatory cell infiltration (score 2); and structural disorganization, intense edema, pyknotic cells, vacuolization, and inflammatory cell infiltration (score 3). We multiplied the two grading scores to obtain damage scores. The average damage score per section was calculated in 4 fields/brain region (including the cortex, striatum, hippocampus, and hypothalamus).

### LFB and cresyl violet staining for evaluating white matter injury and brain cavity volume

LFB staining was used to evaluate the white matter injury with an LFB stain kit (catalog no. ab150675; Abcam) to assess their myelin content. According to the manufacturer's instructions, every tenth section (10 µm thickness, 150 µm apart) was stained with LFB to detect myelin damage. In brief, slides were incubated for 1 h in a preheated solution of LFB (60 °C), differentiated in lithium carbonate solution for 30 s, followed by 70% ethyl alcohol for 30 s, and counterstained in cresyl violet solution for 30–40 s. The slides were coverslipped for the Zeiss microscope (Jena, Germany) examination. All image analyses were performed by a single examiner who was blinded to the sample group. One 0.5 mm^2^ per ROI was selected per section in theCCb and ab of the ipsilateral and contralateral hemispheres. One 0.3 mm^2^ was selected per ROI per section in the ic, laterodorsal thalamic nuclei (LD), and ventroposteromedial (VPM)-ventroposterolateral (VPL) thalamic nuclei. Three brain sections per ROI per rat and ten rats per group were assessed for LFB staining, and the average width (μm) within the ROI was calculated. The threshold intensity was manually set and kept constant to subtract the staining background by using the Color Deconvolution plugin of ImageJ software (RRID: SCR_003073). The LFB positive pixels were used to evaluate the fold change in myelin content described previously^35,36^. TBI caused an expanding cavity in the cortex in a time-dependent manner. For the brain lesion volume measurement, the boundary contours of the contralateral and ipsilateral remaining brain regions were drawn with ImageJ software, and the contours’ enclosed volume was measured with the following formula:

$$\begin{aligned} & {\text{Percentage}}\;{\text{of}}\;{\text{brain}}\;{\text{lesion}}\;{\text{volume}} \\ & \quad = \left( {{\text{contralateral}}\;{\text{brain}}\;{\text{area}} - {\text{ipsilateral}}\;{\text{spare}}\;{\text{brain}}\;{\text{area}}} \right)/{\text{contralateral}}\;{\text{brain}}\;{\text{area}} \times {1}00\% \\ \end{aligned}$$

### IF staining

Before staining, sections were incubated in 0.3% H_2_O_2_ to quench endogenous peroxidases, blocked with 3% goat serum in 0.25% Triton X and 1xPBS, and then incubated with primary antibodies in PBS containing 0.5% normal bovine serum at 4℃ overnight, and the next day with secondary antibodies for 1 h at room temperature. The following antibodies were used: rabbit anti-Caspr (1:200, Abcam Inc., Boston, MA, USA, #ab133634) and mouse anti-ankyrin-G (1:200, Invitrogen, Carlsbad, CA, USA, # 33-8800. RRID: AB_2533145). Caspr + and ankyrin + cells were visualized using Alexa Fluor 488-conjugated goat anti-rabbit IgG (1:400, Invitrogen, CA, USA, #A11008. RRID: AB_143165) and Alexa Fluor 568-conjugated goat anti-rabbit IgG (1:400, Invitrogen, #A11008. RRID: AB_2534072) with excitation and emission wavelengths of 495/525 nm and 578/603 nm respectively. DAPI staining (1:25,000, Thermo Fisher, #62247, excitation 360 nm, emission 460 nm) was performed to visualize nucleated cells. The slides were mounted with mounting medium (Fisher Scientific SP15-500) and examined and photographed under a fluorescence microscope (Zeiss). Images of the yellow-colored immunofluorescence for Caspr/ankyrin-G double-positive cells were captured at 100 × magnification using a microscope system (Axiovision; Zeiss Gmbh, Gottingen, Germany). In each image, immune-positive cells showing staining with a cellular morphology and above background level were manually and exhaustively counted using Axio Vision image analysis software (Zeiss. RRID: SCR_002677). Colocalization of nodal and paranodal proteins at the nodes of Ranvier was assessed using ankyrin-G and Caspr double immunostaining. These fluorescence signal intensities were evaluated in the ROIs of brain regions including the CCb, ab, ic, LD, and VPM-VPL, in brain-injured rats and compared with the ROIs ipsilateral to the injury in sham-operated rats. The area percentage of the Caspr + /ankyrin-G + double immunofluorescence coefficient within each ROI based on an average of three brain sections from each rat was analyzed using Zen software (Zeiss). Subsequently, the values were normalized to those of the sham group.

### Statistical analysis

There were no outliers excluded from the statistical analysis. In any experiment in which the data points were manually obtained, the analysts were masked to the condition. Statistical analyses were performed using GraphPad Prism 8 (GraphPad Software Inc., CA, USA. RRID: SCR_002798). Data are expressed as the mean ± SD. Data were assessed for normality using the Shapiro‒Wilk test. For contusion volume and histological staining analysis, two-way ANOVA was conducted to assess group and time following surgery effects and interactions across each time block. Any significant main effect for group, time or interaction was followed by Tukey’s multiple comparisons post hoc testing. For neurobehavior and MRI analyses, a mixed-effects model two-way repeated-measures ANOVA with Tukey’s multiple comparisons tests were conducted to assess group and time following surgery effects and the interaction between time and group. The "Supplementary Data Table S1" Word file summarized all the datasets' statistical data. *P*-values < 0.05 were considered statistically significant.

### Ethics approval

All animal experiments were conducted under protocols approved by the Institutional Animal Care and Use Committee of Chi Mei Medical Center, Tainan, Taiwan (Approved No.: 105122608) in accordance with the guidelines of the Guide for the Care and Use of Laboratory Animals published by the US National Institutes of Health with due consideration to minimize pain and suffering.

## Results

### rmTBI induced a lower survival rate but a higher contusion volume than TBI at M9

**Figure 2:**
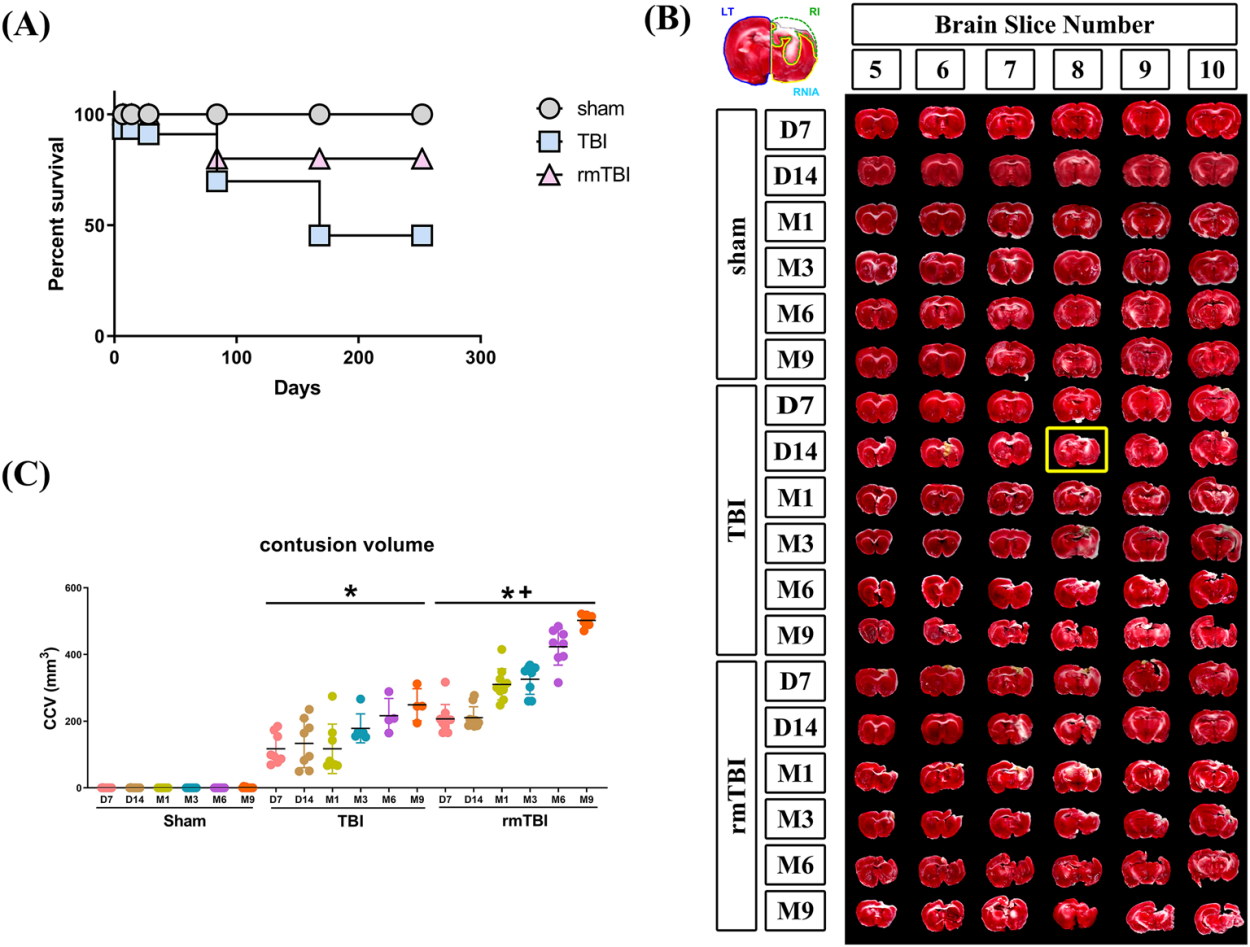
Traumatic brain injury causes both mortality and brain contusion. (**A**) Time-course changes in percentage survival, (**B**) TTC-stained brain sections, and (**C**) cerebral corrected contusion volumes (CCV) values are presented as the mean ± SD. As shown in the left-top of (B), the contusion area (the green dashed line) was calculated by subtracting the non-contusion area in the ipsilateral hemisphere (ipsi., the yellow line) from the area of the whole area in the contralateral hemisphere (contra., the blue line). CCV = 1 mm (thickness of the brain slice) × sum of the contusion area in all brain slices (mm^2^). Compared to the sham group, the TBI and rmTBI groups of rats had significant higher CCVs that persisted for 9 months. *P < 0.05, compared to sham group; and ^+^*P* < 0.05, compared to TBI; *n* = 10–12 (Sham), *n* = 4–8 (TBI), and *n* = 8–10 (rmTBI). Sham: sham operation group; TBI: moderate traumatic brain injury group; rmTBI: repetitive mild traumatic brain injury group; Pre-OP: preoperation.

At the end of M9 (or 270 days), compared to the TBI group rats, the rmTBI group rats had significantly higher values of percentage survival rate (~ 40% vs. 80%, Fig. 2A). Autopsy studies also revealed that TBI group rats displayed more severe subdural and intraparenchymal hematoma formation, loss of ipsilateral cortical tissue, and hippocampal atrophy than did rmTBI group rats (data not shown). In contrast, compared to the TBI group rats, the rmTBI group rats had a significantly higher corrected contusion volume (CCV) (~ 400 mm^3^ vs. ~ 100 mm^3^, Fig. 2B,C), and a significantly higher incidence rate of epilepsy syndromes (8 in 10 rmTBI rats vs. 5 in 10 TBI rats) at 9 months post injury.

### rmTBI induced more significant neurobehavioral deficits than TBI

**Figure 3:**
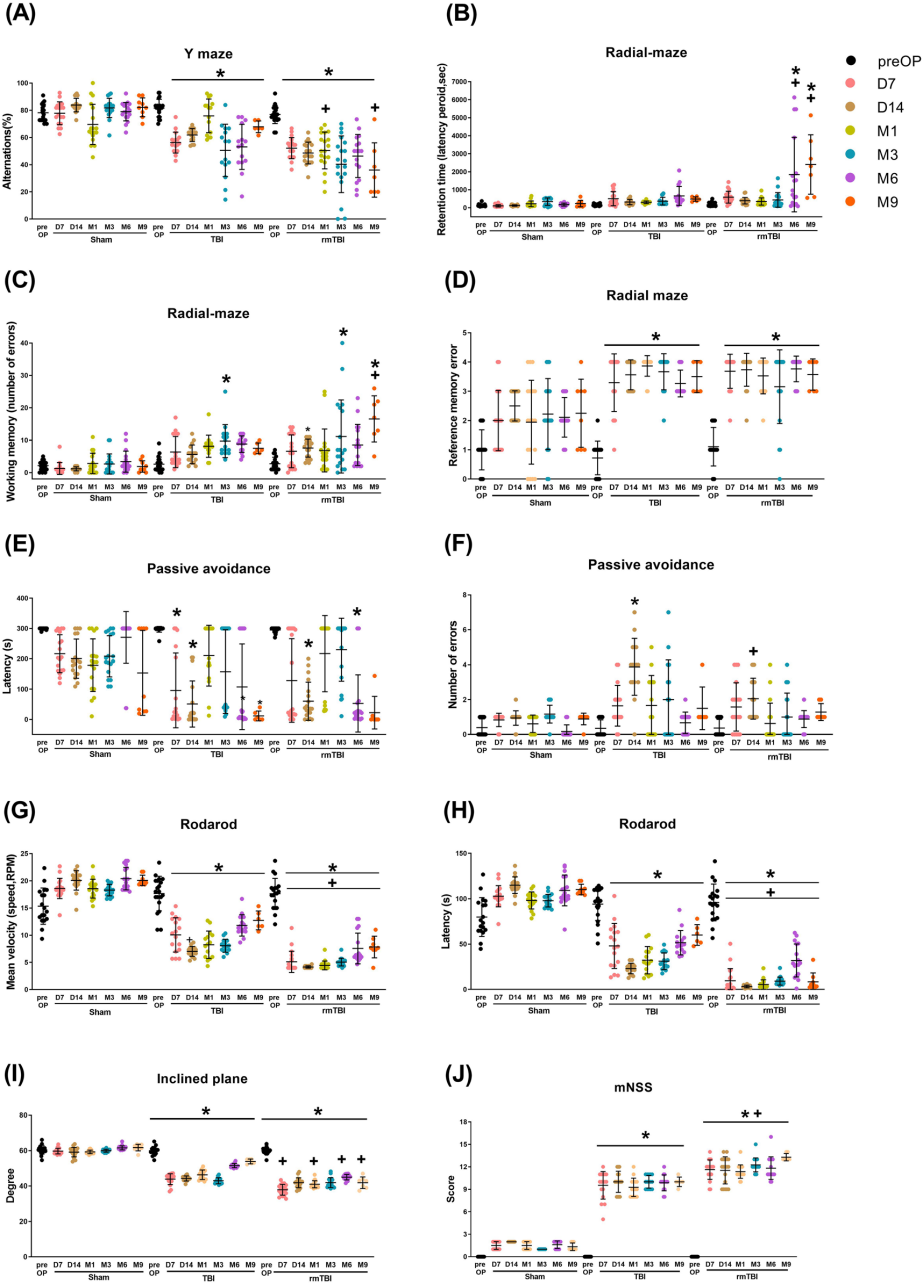
Traumatic brain injury causes neurobehavioral disorders. Neurological functions evaluation after TBI. The behavioral test battery consisted of three memory tests (Y maze, radial maze, and passive avoidance), two motor tests (rotarod and inclined plane), and one modified neurological severity score (mNSS) were performed before the operation (pre-OP) and at 7 days to 9 months after surgery. (**A**) The TBI and rmTBI rats showed a lower level of alternation at different times on the Y maze after neurotrauma. Compared to the sham group, the (**B**) retention time (latency period, seconds), (**C**) working memory errors, and (**D**) reference memory errors on the radial-arm maze test were significantly increased in both TBI and rmTBI groups. On the passive avoidance test, the retention latency (**E**) was significantly shorter, and the number of errors (**F**) was significantly higher in TBI and rmTBI rats than that in sham animals from day 7 to month 9. The TBI and rmTBI groups had significantly decreased (**G**) mean velocity (speed, RPM) and (**H**) the latency (s) in the rotarod test compared to the sham group. (**I**) The inclined plane was used to measure limb strength. The higher the maximal angle a rat could hold on to the plane, the less severe the injury was. Compared to the sham group, the TBI and rmTBI groups of rats had a significant decrease in the maximal angle. (**J**) mNSS was used to assess neurological deficits. The higher the score is, the more severe the injury. Compared to the sham group, the TBI and rmTBI groups of rats had a significantly higher mNSS. Data are presented as mean ± SD (*n* = 20 of Sham, *n* = 8–16 of TBI, and *n* = 16–20 of rmTBI). *P < 0.05, compared with the sham group. ^+^*P* < 0.05, compared with the TBI group.

A behavioral test battery consisted of three cognitive tests (Y-maze, radial maze, and passive avoidance), two motor tests (rotarod and incline plane), and one neurological severity score assay were performed to evaluate cognitive, motor, and neurological function, respectively (Fig. 3). Compared to TBI group rats, rmTBI group rats had significantly greater cognitive, motor, and neurological deficits when evaluated within a period of 9 months (Fig. 3).

#### Y-maze test

The TBI and rmTBI rats were impaired in the Y-maze test compared to the sham group during the whole test period, starting at D7 of post-surgery (Fig. 3A). Nine months following TBI, rat displayed deficits in working memory (e.g., decreased percentage of alterations in Y-maze test. Analysis with a mixed effects two-way ANOVA model found significant effects of the group [*F*(2,319) = 170.1, *P* < 0.0001], time [*F*(6,319) = 20.38, *P* < 0.0001], and group × time interaction [*F*(12,319) = 10.83, *P* < 0.0001]. Post-hoc comparisons with Tukey’s multiple comparison test indicated significant decrease in alterations in rmTBI on month 1 (M1) and months 9 (M9) compared to TBI M1 and M9 values (*P* < 0.05).

#### Radial maze test

Compared to sham rats or TBI rats, rmTBI rats had a significant increase in retention time (Fig. 3B) and working memory errors (Fig. 3C) in the radial maze test. For retention time, two-way ANOVA for mixed effects of group [*F*(2,319) = 8.68, *P* < 0.0001], time [*F*(6,319) = 34.53, *P* < 0.0001], and group × time interactions [*F*(12,319) = 8.68, *P* < 0.0001] were significant. Rats in rmTBI alone exhibited increased M6 and M9 retention time values (compared with sham or TBI, *P* < 0.05). For the numbers of working memory errors, two-way ANOVA for mixed effects of group [*F*(2,319) =57.42, *P* < 0.0001], time [*F*(6,319) = 9.497, *P* < 0.0001], and group × time interactions [*F*(12,319) = 3.211, *P* = 0.0012] were significant. Post-hoc comparisons with Tukey’s multiple comparison test indicated a significant increase in working memory errors in TBI on M3 and rmTBI on days 14 (D14), M3, and M9 compared to Sham D14, M3, and M9 values (*P* < 0.05). Both TBI (*P* < 0.05, all time points) and rmTBI (*P* < 0.05, except M6) rats had significant increases in values of reference memory errors (Fig. 3D) in comparison with sham rats.

#### Passive avoidance test

For 9 months of observation, the Sham group of rats did not show any learning and memory deficits. By using mixed effects repeated measures two-way ANOVA analysis on the latency of passive avoidance revealed a significant main effect for group [*F*(2,266) = 26.89, *P* < 0.0001], for time [*F*(6,319) = 30.64, *P *< 0.0001] and group × time interactions [*F*(12,319) = 5.687, *P *< 0.0001] (Fig. 3E). Compared to sham group rats, both TBI (*P* < 0.05, values of D7 and D14) and rmTBI (*P* < 0.05, values of D14 and M6) group had a significant decrease in latency of passive avoidance test. At D14, TBI and rmTBI rats had a statistically significant increase in the number of errors in the passive avoidance test (Fig. 3F) in comparison with the sham group (*P* < 0.05).

#### Rotarod test

Both TBI and rmTBI rats were impaired in the rotarod test compared to sham group rats during the whole test period, starting at D7 of post-surgery (Fig. 3G,H). Nine months post-TBI, rats had a statistically significant deficit in motor function (e.g., decreased mean velocity [group factor: *F*(2,319) = 612, *P* < 0.0001; time factor: *F*(6,319) = 78.11, *P* < 0.0001; group × time: *F*(12,319) = 48.9, *P* < 0.0001] and decreased latency in rotarod test [group factor: *F*(2,319) = 705.9, *P* < 0.0001; time factor: *F*(6,319) = 77.64, *P* < 0.0001; group × time: *F*(12,319) = 52.93, *P* < 0.0001]).

#### Inclined plane test

Compared to the sham group, the TBI and rmTBI groups had a significant decrease in the maximal angle on the inclined plane at all observation time points (Fig. 3I). Analysis with a mixed effects 2-way ANOVA model found a significant interaction between time and group [*F*(12,319) = 69.54, *P* < 0.0001]. Significant effects of time (*P* < 0.0001) and group (*P* < 0.0001) were noted. Tukey’s multiple comparison test indicated a significant decrease in maximum angle in rmTBI D7, M1, M6, and M9 compared to TBI D7, M1, M6, and M9 values (*P* < 0.05).

#### mNSS test

The mNSS serves as a criterion for the neurological assessment of movement, reflex, sensation, and balance in rats after surgery. There was a significant increase in mNSS after TBI and rmTBI in comparison to the sham group at all observation time points (Fig. 3J). Two-way ANOVA revealed a significant interaction between the group factor and time condition [*F* (12,319) = 94.69; *P* < 0.0001], a significant effect of group [*F*(2,319) = 1508; *P* < 0.0001], and a significant effect of time [*F*(6,319) = 541.5; *P* < 0.0001]. Post-hoc comparisons with Tukey’s multiple comparison test indicated a significant increase in neurological deficits in rmTBI compared to TBI values (*P* < 0.05).

### rmTBI induced greater cortical damage than TBI

**Figure 4:**
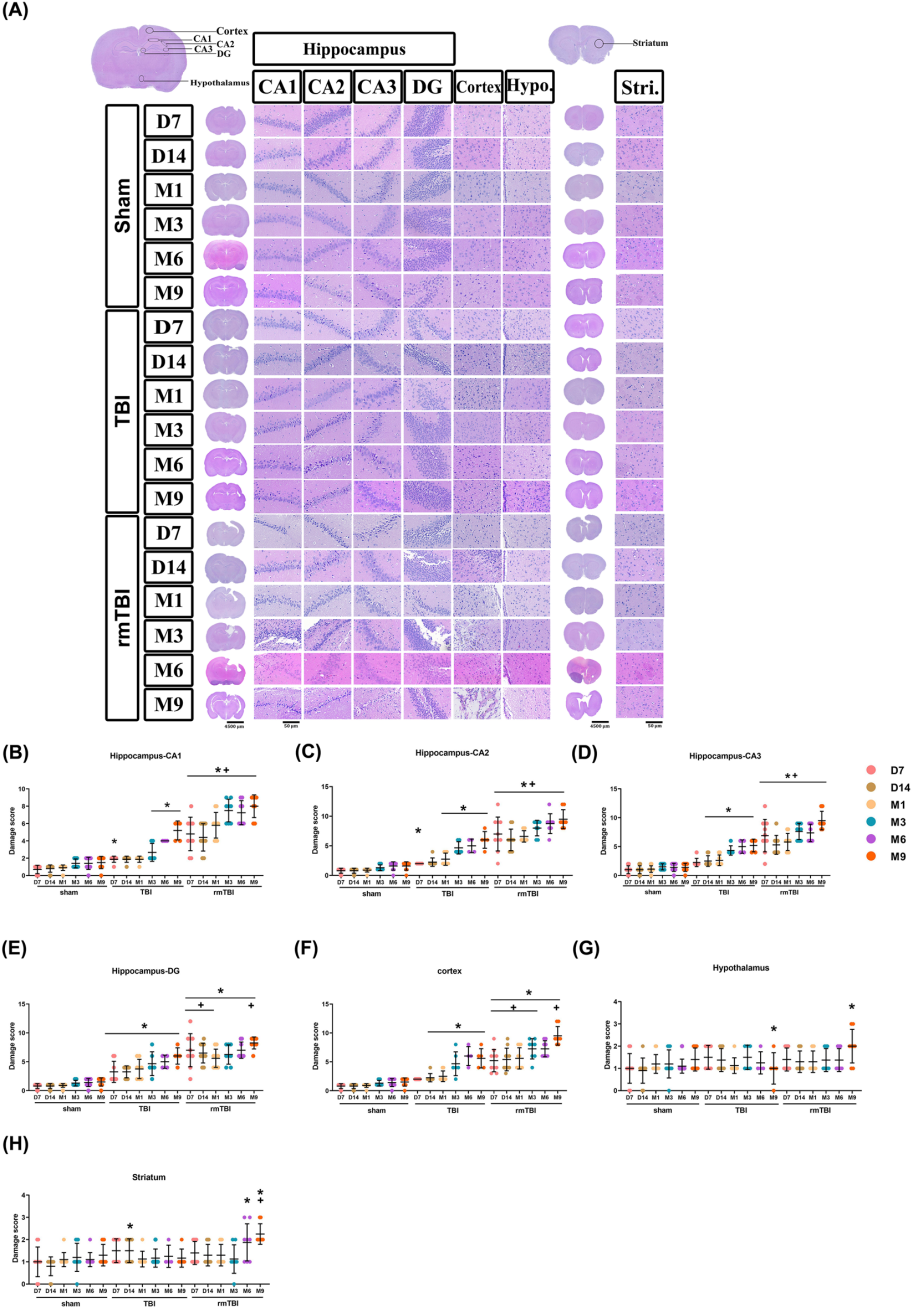
Traumatic brain injury causes hippocampal and cortical damage. Time-dependent progression of histopathological changes after TBI and rmTBI. (**A**) H&E-stained brain sections showed cell shrinkage with dark-stained pyknotic nuclei and perineuronal vacuolization in the hippocampus, cortex, hypothalamus, and striatum. The boxed areas in the full-size brain image on the top left graphic are magnified in the representative image. Scale bar, 100 μm. Bar graph representing the mean ± SD (*n* = 10 of Sham, *n* = 4–8 of TBI, and *n* = 8–10 of rmTBI) of neuronal damage scores in (**B**) hippocampus-CA1 region, (**C**) hippocampus-CA2 region, (**D**) hippocampus-CA3 region, (**E**) hippocampus-DG region, (**F**) cortex, (**G**) hypothalamus, and (**H**) striatum at different time points after TBI for each experimental group of rats. *P < 0.05, compared with the sham group. ^+^*P* < 0.05, compared with the TBI group.

H&E staining (Fig. 4A) revealed that, compared to the sham group rats at M3 to M9, the TBI group at M3 to M9 and the rmTBI group at D7 to M9 had significantly higher values of damage scores in different brain regions, including: including the hippocampal CA1 (Fig. 4B), CA2 (Fig. 4C), CA3 (Fig. 4D), DG (Fig. 4E), cortex (Fig. 4F), and striatum (Fig. 4H). In the hypothalamus region, we found no statistically significant difference of damage scores among the three experimental groups (Fig. 4G). The rmTBI group at D7 to M9 had significantly higher hippocampal and cortical damage scores than the TBI group rats at D7 to M9 (Fig. 4).

**Figure 5:**
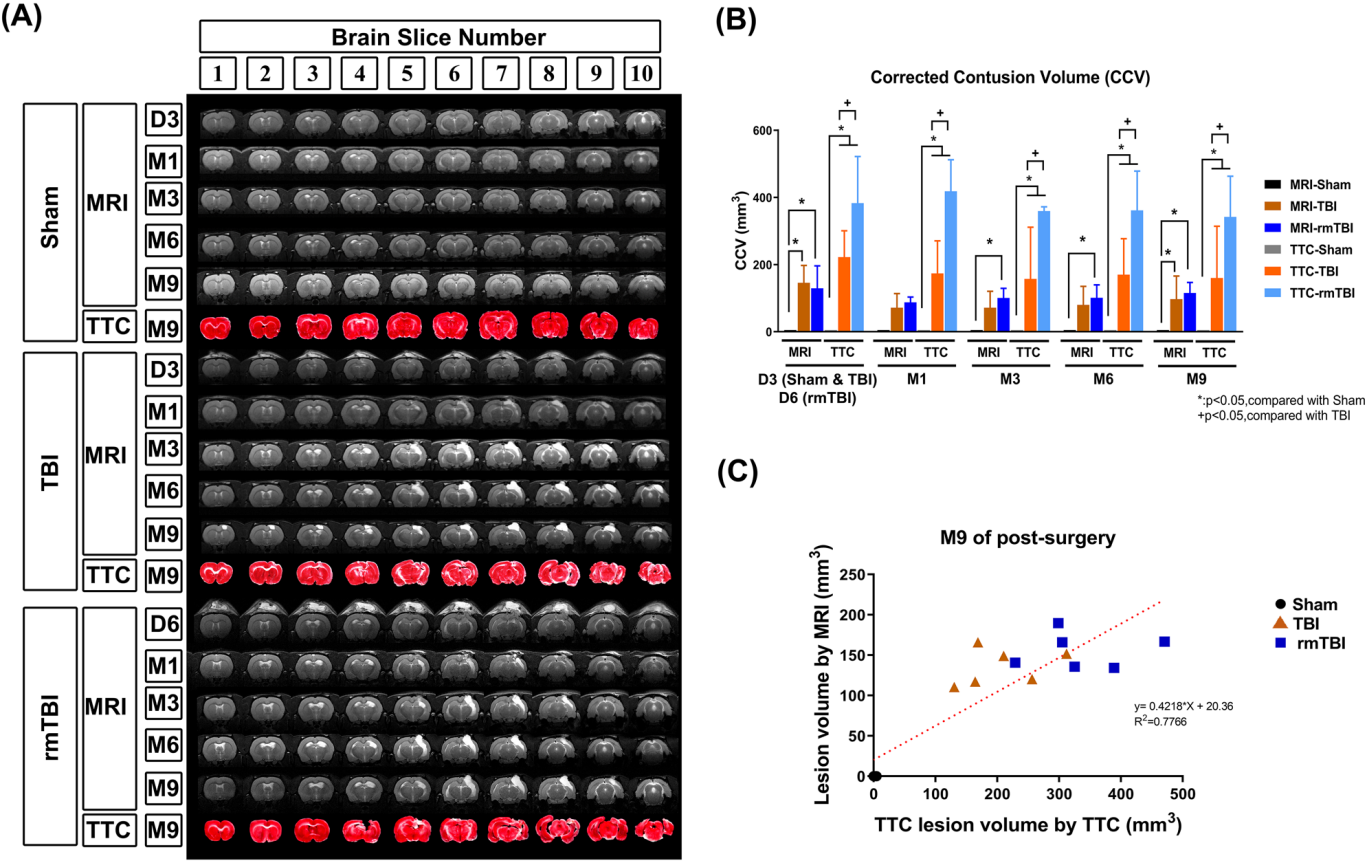
Spatiotemporal variation in contusion volume by using T2WI was correlated with TTC staining and behavioral tests in TBI rats. (**A**) Representative coronal T2WI images of a sham, a TBI, and a rmTBI rat brain from day 3 to month 9 after surgery. The brains were removed after the last MRI procedure and stained with TTC to verify the contusion volume verification. (**B**) At different time points, the corrected lesion volumes (mm^3^) were obtained from T2WI to those obtained from TTC stained brain sections in a separate group of sham, TBI, and rmTBI. Data are presented as the mean ± SD (*n* = 12 of Sham,* n* = 9 of TBI, and *n* = 8 of rmTBI). *P < 0.05, compared with the sham group. ^+^*P* < 0.05, compared with the TBI group. (**C**) Correlation coefficient analysis between neurological function score and contusion volume (9 months after surgery).

Both T2-weighted MRI (T2WI) and TTC staining (Fig. 5A) confirmed that compared to the sham group rats at D3 to M9, the TBI group at D3 to M9 and the rmTBI group at D3 to M9 had significantly higher CCV values (Fig. 5B).

Again, compared to TBI, rmTBI had significantly higher values of cerebral lesion volume by T2WI and TTC staining (Fig. 5B). The linear regression correlation test showed a positive correlation between cerebral contusion volume measurements using MRI and TTC of month 9 data in the TBI or rmTBI group (Fig. 5C). The time-dependent changes in T2WI, CE-T1WI, and DTI whole-brain images from sham, TBI, and rmTBI rats are also available in video format (Supplementary Video 1).

### Both TBI and rmTBI equally disrupted blood‒brain barrier permeability

**Figure 6:**
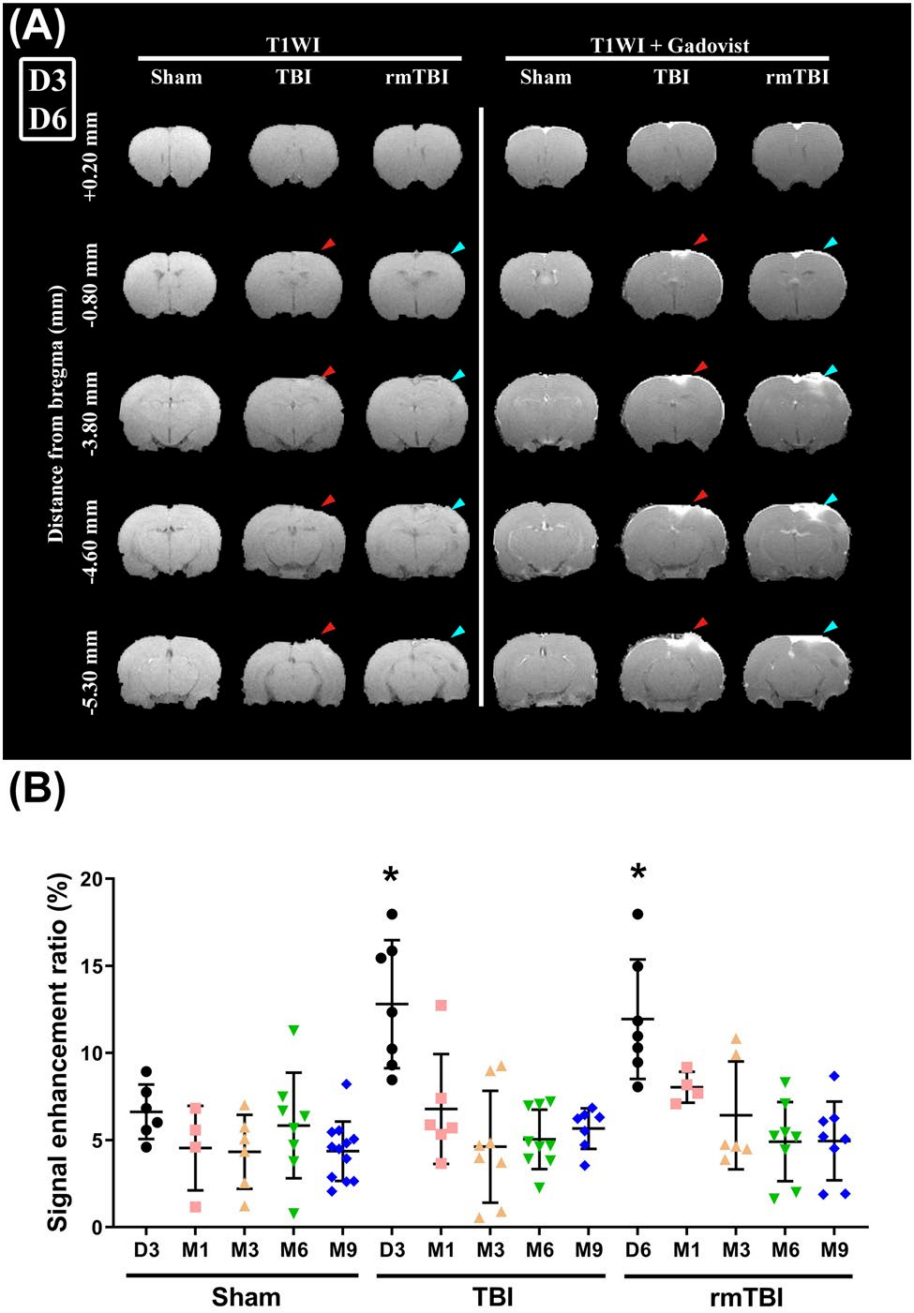
TBI causes blood–brain barrier disruption with contrast-enhanced T1-weighted imaging (CE-T1WI). (**A**) Representative CE-T1WI images in a sham, a TBI, and a rmTBI rat from at D3 (Sham group and TBI group) or D6 (rmTBI group) after surgery. (**B**) Mean ratios of T1 enhancement were significantly larger in the TBI and rmTBI rats than that in the Sham rats at day 3 or day 6 after surgery. Data are presented as the mean ± SD (*n* = 12 of Sham,* n* = 9 of TBI, and *n* = 8 of rmTBI ). *P < 0.05, compared with the sham group.

As shown in the T1WI images in Fig. 6A, compared to the sham group at D3, the TBI group and the rmTBI group at D6 had significantly higher BBB disruption measurements using CE-T1WI (Fig. 6B). However, the signal enhancement ratios of rmTBI were not significantly different from those of TBI group rats (Fig. 6).

### rmTBI induced more severe myelin and axon pathology than TBI

**Figure 7:**
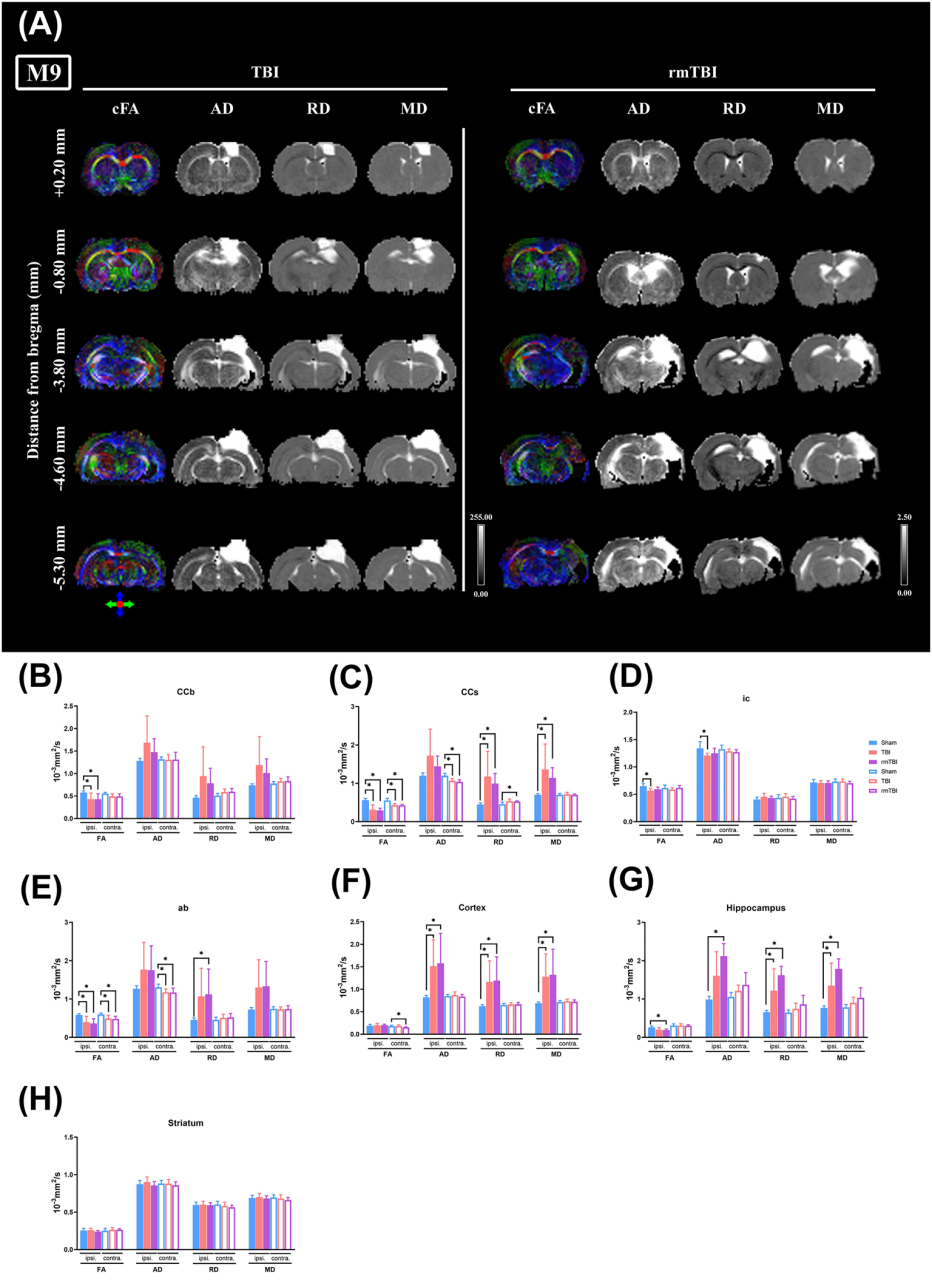
TBI causes alterations in DTI parameters. Coronal images of the diffusion parameter map (bregma + 0.20 mm to − 5.3 mm) were obtained from a TBI and a rmTBI rat at month 9 (M9) post-injury. (**A**) Fractional anisotropy (FA), mean diffusivity (MD), radial diffusivity (RD), and axial diffusivity (AD) maps are shown, and the data are summarized in Table 1 and Supplementary file. The cFA brain map with direction-encoded color maps was based on the extracted direction: red was for medial–lateral (x-axis), green was for rostral-caudal (y-axis), and blue was for dorsal–ventral (z-axis). The grayscale indicates FA values between 0 (black) and 1 (white) and AD, RD, and MD values between 0 (black) and 255 (white). The four indices FA, AD, RD, and MD for each brain structure are analyzed at M9 of post-injury, including (**B**) CCb, (**C**) CCs, (**D**) ic, (**E**) ab, (**F**) cortex, (**G**) hippocampus, and (**H**) striatum. Blue bars represent the Sham group (n = 12), orange bars TBI group (n = 9), and purple bars rmTBI group (n = 8). *P < 0.05, compared with the sham group. The “Supplementary Data" file shows DTI brain maps at the other time points and the statistical analysis data.

DTI noninvasively detects myelin and axon pathology and quantifies the directionality of water diffusion, which moves preferentially along white matter fibers. A number of measures can be derived from DTI, including AD, RD, and MD, and FA. DTI observations from D3 to M9 postinjury revealed that, for the DTI measure of FA, two-way ANOVA detected a significant effect of injury in the ipsilateral ab from M1 to M9, ipsilateral CCs from M3 to M9, ipsilateral ic from M6 to M9, ipsilateral hippocampus at M1 and M9, contralateral CCb from M6 to M9, contralateral CCs from M3 to M9, contralateral ab at M3 and M9, and contralateral cortex at M3 and M9, indicating that FA was reduced in TBI and/or rmTBI rats compared to the sham group (Fig. 7, Table 1 and Supplementary Figures S3–S6 and Supplementary Table S2–S5). For the DTI measure of AD, two-way ANOVA detected a significant effect of injury in the ipsilateral hippocampus from M1 to M9, ipsilateral cortex from M6 to M9, contralateral CCs from M6 to M9, and contralateral ab at M9, indicating that AD was enhanced or reduced in TBI and/or rmTBI rats compared to sham rats. For the DTI measure of RD, two-way ANOVA detected a significant effect of injury in the ipsilateral CCs from M3 to M9, ipsilateral CCb at M3, ipsilateral cortex from M6 to M9, ipsilateral hippocampus from M3 to M9, and contralateral CCs from M3 to M9, indicating that RD was enhanced in TBI and/or rmTBI rats compared to sham rats. For the DTI measure of MD, two-way ANOVA detected a significant effect of injury in the ipsilateral hippocampus at M1, ipsilateral CCs, ipsilateral cortex, and ipsilateral hippocampus at M9, indicating MD was enhanced in TBI and/or rmTBI rats compared to sham rats. Figure 7 represents and quantifies the color-coded FA (cFA), AD, RD, and MD in brain sections from bregma + 0.2 to − 5.3 mm at M9 postinjury. The DTI brain sections at D3, M1, M3, and M6 are represented in the "Supplementary Data" file. The overall MRI brain sections were merged and dynamically expressed in the "Supplementary Video-1" file.

**Table 1:** Fractional anisotropy (FA) and axial diffusivity (AD), radial diffusivity (RD), mean diffusivity (MD) from ipsilateral and contralateral hemisphere in sham control (n = 6) and rats after mTBI (n = 6) or rTBI at month 9 (n = 6) obtained using in vivo DTI.

	FA			AD			RD			MD		
	Sham	mTBI	rTBI	Sham	mTBI	rTBI	Sham	mTBI	rTBI	Sham	mTBI	rTBI
Ipsilateral												
WM:												
CCb	0.54 ± 0.01	0.43 ± 0.03*	0.39 ± 0.03*	1.28 ± 0.02	1.68 ± 0.20*	1.47 ± 0.11	0.57 ± 0.04	0.95 ± 0.12	1.13 ± 0.11*	0.85 ± 0.04	1.21 ± 0.12	1.39 ± 0.12*
CCs	0.56 ± 0.02	0.33 ± 0.03*	0.33 ± 0.02*	1.21 ± 0.02	0.99 ± 0.03*	0.99 ± 0.02	0.45 ± 0.02	0.59 ± 0.02*	0.60 ± 0.02*	0.70 ± 0.01	0.72 ± 0.02*	0.73 ± 0.02*
ic	0.60 ± 0.01	0.54 ± 0.01*	**0.62 ± 0.02 + **	1.34 ± 0.03	1.30 ± 0.02	1.27 ± 0.01	0.46 ± 0.02	0.51 ± 0.01	**0.41 ± 0.01 + **	0.75 ± 0.02	0.77 ± 0.01	0.70 ± 0.01
ab	0.54 ± 0.02	0.46 ± 0.03	0.43 ± 0.02*	1.23 ± 0.03	1.16 ± 0.03	1.12 ± 0.03	0.47 ± 0.02	0.53 ± 0.04	0.54 ± 0.03	0.73 ± 0.02	0.74 ± 0.02	0.74 ± 0.02
GM:												
Cortex	0.19 ± 0.01	0.20 ± 0.01	0.20 ± 0.01	0.82 ± 0.01	1.51 ± 0.20*	1.24 ± 0.24*	0.61 ± 0.01	1.16 ± 0.16*	1.19 ± 0.19*	0.69 ± 0.01	1.28 ± 0.17*	1.32 ± 0.20*
Hippo	0.23 ± 0.01	0.19 ± 0.01*	0.19 ± 0.01*	0.94 ± 0.02	1.45 ± 0.02*	1.90 ± 0.13* +	0.65 ± 0.01	1.10 ± 0.18*	1.45 ± 0.11* +	0.74 ± 0.01	1.22 ± 0.19*	1.60 ± 0.11* +
Stria	0.24 ± 0.01	0.24 ± 0.01	0.24 ± 0.01	0.86 ± 0.01	0.97 ± 0.05*	0.95 ± 0.03	0.60 ± 0.01	0.68 ± 0.04*	0.68 ± 0.02*	0.69 ± 0.01	0.77 ± 0.04*	0.77 ± 0.02*
Contralateral												
WM:												
CCb	0.56 ± 0.01	0.38 ± 0.04*	0.36 ± 0.04*	1.31 ± 0.02	1.30 ± 0.04	1.31 ± 0.09	0.54 ± 0.03	1.26 ± 0.21*	1.33 ± 0.15*	0.83 ± 0.03	1.52 ± 0.20*	0.83 ± 0.03*
CCs	0.54 ± 0.02	0.30 ± 0.05*	0.24 ± 0.02*	1.19 ± 0.02	1.55 ± 0.21	1.52 ± 0.14	0.47 ± 0.02	1.07 ± 0.21	1.11 ± 0.11	0.70 ± 0.01	1.23 ± 0.21	1.25 ± 0.12
ic	065 ± 0.01	0.55 ± 0.02*	0.58 ± 0.03*	1.33 ± 0.04	1.20 ± 0.01*	**1.34 ± 0.03 + **	0.40 ± 0.01	0.47 ± 0.03	0.49 ± 0.04*	0.71 ± 0.02	0.71 ± 0.02	0.78 ± 0.03
ab	0.55 ± 0.01	0.32 ± 0.03*	0.29 ± 0.03*	1.23 ± 0.03	1.54 ± 0.21	1.77 ± 0.29*	0.47 ± 0.02	0.99 ± 0.19*	1.23 ± 0.26*	0.72 ± 0.02	1.17 ± 0.19*	1.41 ± 0.27*
GM:												
Cortex	0.18 ± 0.01	0.18 ± 0.07	0.15 ± 0.01	0.85 ± 0.01	0.87 ± 0.02	0.83 ± 0.02	0.65 ± 0.01	0.66 ± 0.01	0.66 ± 0.02	0.71 ± 0.01	0.73 ± 0.02	0.72 ± 0.02
Hippo	0.26 ± 0.01	0.25 ± 0.01	0.25 ± 0.01	1.00 ± 0.02	1.06 ± 0.03	1.16 ± 0.07	0.66 ± 0.01	0.71 ± 0.03	0.78 ± 0.05	0.77 ± 0.02	0.83 ± 0.03	0.91 ± 0.06
Stria	0.23 ± 0.01	0.23 ± 0.01	0.24 ± 0.01	0.86 ± 0.01	0.85 ± 0.02	0.84 ± 0.01	0.61 ± 0.01	0.59 ± 0.02	0.58 ± 0.01	0.69 ± 0.01	0.68 ± 0.02	0.67 ± 0.01

Statistical significance are *P < 0.05 comparing the same hemisphere in sham control and rats after TBI (one-way ANOVA with Tukey's multiple comparisons test) and + p < 0.05 comparing the same hemisphere in rTBI group and mTBI group. CCb, Body of the corpus callosum; CCs, Splenium of the corpus callosum; ic, Internal capsule; ab, Anterior commissure; Hippo, Hippocampus; Stria.: Striatum; WM, White matter; GM, Gray matter. Bold text for significantly increased values and significantly decreased values.

In the white matter of the contralateral hemisphere, compared to the sham group rats, the TBI and rmTBI group rats had significantly lower values of FA (CCb, CCs, ab) and AD (CCs, ab) but significantly higher values of RD (CCb, CCs) at M6 and M9 of postinjury (Fig. 7 and Supplementary Fig. S6).

In the gray matter of the ipsilateral hemisphere, compared to the sham group rats, the TBI and rmTBI rats had significantly lower values of FA in the hippocampus at M1 and M9 of postinjury, and higher values of AD (cortex, hippocampus), RD (cortex, hippocampus) and MD (cortex, hippocampus) at M1 to M9 of postinjury (Fig. 7 and Supplementary Fig. S3–S6). In fact, a significant correlation between the lesion volume (by T2WI) and DTI values (such as FA, RD, and MD) in CCs and ab regions of both TBI and rmTBI group rats evaluated during 1–9 months after injury was noted (please see supplementary Table S6).

**Figure 8:**
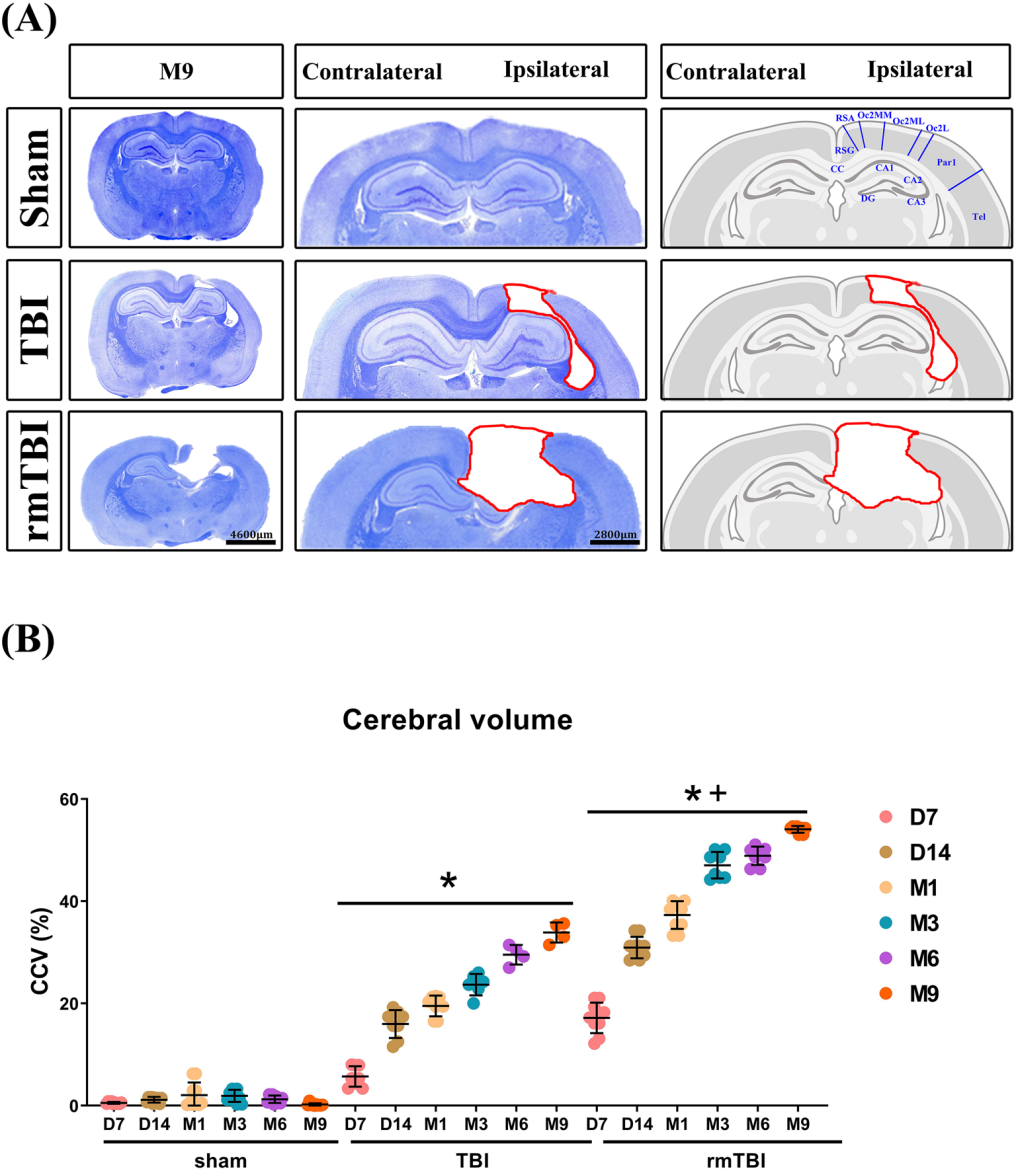
TBI causes brain cavity formation and white matter thickness reduction. Luxol fast blue (LFB) and cresyl violet (or Nissl) staining show the temporal progression of the brain lesion. LFB staining was used to identify myelin. Cresyl violet was used to stain neuronal cell bodies, processes, and Nissl bodies. (**A**) Representative LFB with Nissl staining from coronal sections at 9 months (M9) post sham operation, post TBI, and post rmTBI. For all time points in coronal sections from D3 to M9 of each group, please see Figure S7 in the “Supplementary Data” file. (**B**) Quantification of brain cavity volume (lesion volume) in different severities of TBI. Data are presented as mean ± SD (*n* = 12 of Sham,* n* = 9 of TBI, and *n* = 8 of rmTBI). *P < 0.05, compared with the sham group. ^+^*P* < 0.05, compared with the TBI group.

**Figure 9:**
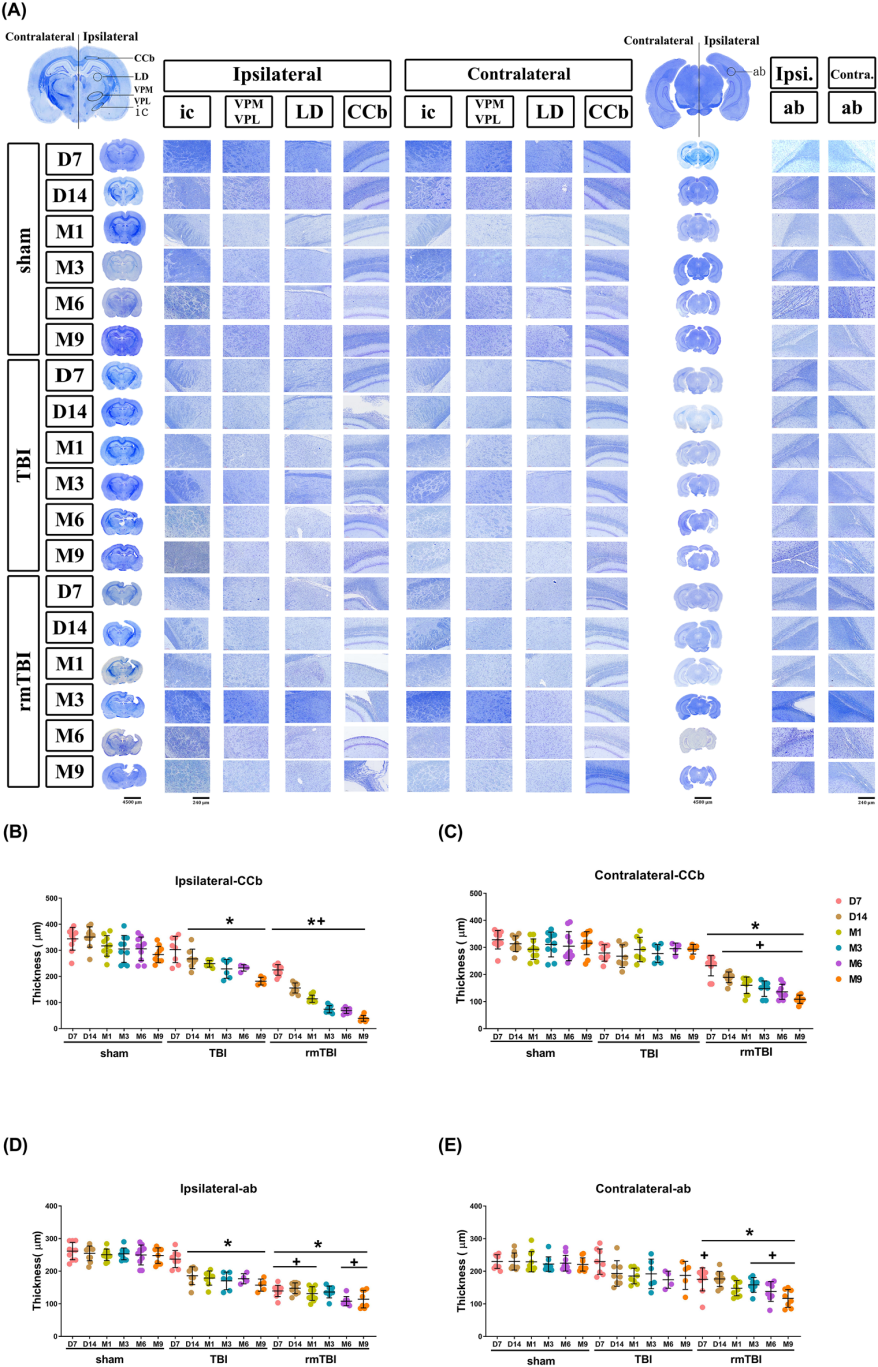
TBI causes structural abnormalities in white matter. Temporal and spatial patterns of white matter injury after TBI. (**A**) Luxol fast blue with Nissl stained white matter in the ipsilateral and contralateral regions of brain slices at 7 days to 9 months after the operation. The circled regions of interest (ROIs) included the internal capsule (ic), ventroposteromedial (VPM), ventroposterolateral (VPL), laterodorsal thalamic nuclei (LD), body of the corpus callosum (CCs), and angular bundle (ab). The selected areas in full-size brain images are magnified to the representative image. Quantification of white matter thickness in the (**B**) ipsilateral CCs, (**C**) contralateral CCs, (**D**)ipsilateral ab, and (**E**) contralateral ab brain regions. Data are represented as the means ± SD (*n* = 4–10). *P < 0.05, compared with sham. ^+^*P* < 0.05, compared with TBI.

LFB and cresyl violet staining revealed that compared to the TBI group rats, the rmTBI group at D14 to M9 had significantly higher values of brain lesion volume (Fig. 8A,B, and Supplementary Figure S7 for all time points stained brain slices) but significantly lower values of the thickness of CCb, CCs, ic, and ab of the ipsilateral hemisphere (Fig. 9A–E). The rmTBI group had significantly higher values of brain lesion volume and lower white matter thickness values than did the TBI group. In particular, white matter thickness in the ipsilateral CCb (Fig. 9B), contralateral CCb (Fig. 9C), ipsilateral ab (Fig. 9D), and contralateral ab (Fig. 9E) was significantly reduced. However, the LD, VPL, and VPM were not significantly affected by TBI (data not shown).

**Figure 10:**
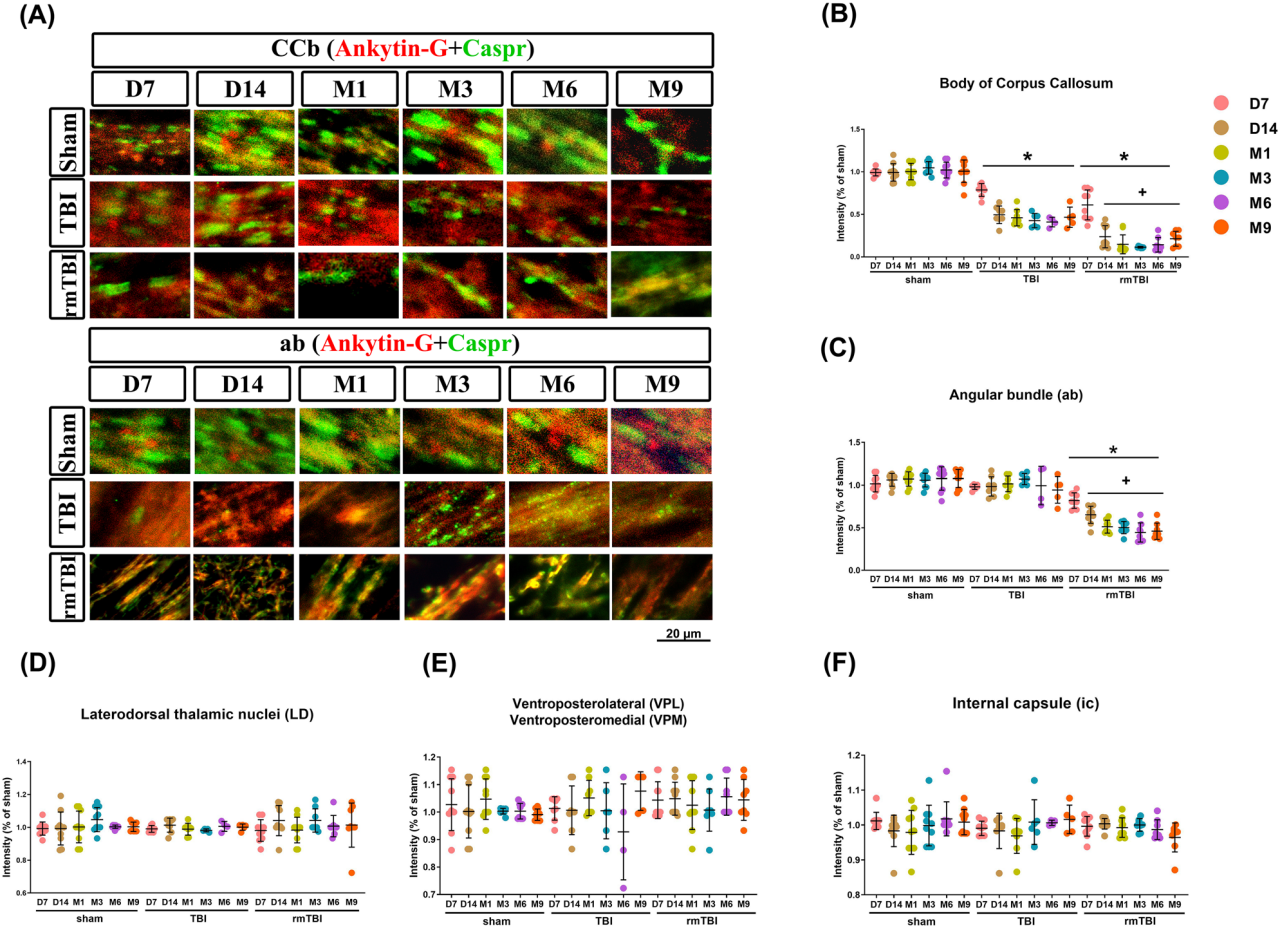
TBI causes structural abnormalities at the nodes of Ranvier. Coclustering of ankyrin G (AnkG, nodal protein) and contactin-associated protein (Caspr, paranodal protein) at the nodes of Ranvier after TBI. (**A**) Representative images of sections from sham, TBI, and rmTBI groups. Colocalization puncta intensity of AnkG and Caspr signals in (**B**) CCs, (**C**) LD, (**D**) VPL and VPM, (**E**) ic, and (**F**) ab of ipsilateral brain regions. Data are represented as the means ± SD (*n* = 4–10). *P < 0.05, compared with the sham group. ^+^*P* < 0.05, compared with the TBI group.

In a myelinated axon at the node, ankyrin G (AnkG, a nodal protein) and contactin-associated protein (Caspr, a paranodal protein) are located at the nodal axolemma and paranodal loops and junctions, respectively^37^. As compared to those of sham controls, the TBI group had a significantly lesser puncta intensity of coclustering of both AnkG and Caspr (Fig. 10A) at the node of Ranvier of both CCb (Fig. 10B) and ab (Fig. 10C), but not of the LD, VPL and VPM, and ic (Fig. 10D–F) after TBI. Again, the rmTBI group had a significantly lesser intensity of coclustering both AnkG and Caspr protein than did the TBI at D14 to M9 (Fig. 10).

## Discussion

In this study, MRI/DTI, LFB staining, cresyl violet staining (alternatively known as Nissl stain), and Caspr and Ankyrin-G staining were used to assess the microstructures, myelin structures, neuronal cell bodies, and nodes of Ranvier and axon initial segments, respectively. A key objective of correlating histological findings with DTI-derived metrics is to validate and interpret the DTI signals, thereby bridging macroscopic imaging manifestations with underlying molecular and cellular phenomena. Notably, 9 months of post-TBI and rmTBI, regions within the CCs, cortex, and hippocampus displayed diminished myelin, as evidenced by attenuated LFB staining, consistently exhibited decreased FA and elevated MD values on DTI (refer to Fig. 7C, F, and G). The nodes of Ranvier, characterized as gaps within the myelin sheath, facilitate the rapid propagation of electrical impulses along axons. Perturbations in the structural or functional attributes of these nodes, such as the diminished presence or altered distribution of Caspr or Ankyrin-G, can adversely affect white matter integrity. Given that FA metrics from DTI inherently capture white matter coherence, deviations in Caspr and Ankyrin-G staining patterns are postulated to influence FA readings. For example, at the 9-months mark following TBI and rmTBI, pronounced decreases in Caspr and Ankyrin-G co-clustering staining in the CCb and ab regions, indicative of compromised nodes of Ranvier, corresponded with decreased FA measurements within related white matter tracts (see Figs. 7B, E, and 10B, C). Establishing such correlations between DTI parameters and Caspr and Ankyrin staining deepens our comprehension of micro-to-macro structural transitions, elucidating the pathophysiological mechanisms in TBI and the foundational bases for observed changes in DTI metrics.

A recent study demonstrated that combining multiple indices of DTI can better differentiate patients with TBI from healthy subjects^38^. In a rat model, the combination of diffusion MRI (dMRI) can provide a more complete insight into the microstructural alterations in white and gray matter after mild TBI, which may aid diagnosis and prognosis following a mild brain injury^39^. An experimental mild brain injury revealed changes in DTI parameters associated with inflammatory processes, reduced myelin density and/or axonal change in white and gray matter areas^39,40^. In this study, we utilized MRI-based DTI to noninvasively investigate the microstructural attributes of white matter tracts within the brain. Simultaneously, we evaluate myelin structures, neuronal cell bodies, and the nodes of Ranvier and axonal initial segment using Caspr and Ankyrin-G assessment, respectively.

DTI-MRI measurements record signals from the motion of water molecules in small imaging volumes (voxels). Their sensitivity is associated with the influence of microstructural alterations impacted by TBI^41^. T2-weighted MRI scans detected increased extracellular fluid in TBI brain^42^. Indeed, our present results also showed that compared to sham rats, the volumes of blood–brain barrier disruption evaluated using contrast-enhancement T1-weighted imaging with Gadovist ( a gadolinium-based MRI contrast agent) were significantly more prominent in both the TBI and rmTBI rats on day 3, but not other time points after LFP. DTI noninvasively detects myelin and axon pathology and quantifies the directionality of water diffusion, which is preferentially along white matter fiber. A number of measures can be derived from DTI, including AD, RD, MD, and FA^43,44^. A lower FA signifies less anisotropic diffusion as well as lower microstructural integrity^45,46^. Additionally, AD and RD may provide additional information on the underlying mechanisms of white matter integrity. Myelin breakdown has been associated with increased RD, while axonal damage is reflected by AD changes^47,48^. As shown in the present results, our DTI observations from D3 to M9 postinjury revealed that reduced FA was noted in the ipsilateral ab from M1 to M9, ipsilateral CCs from M3 to M9, ipsilateral ic from M6 to M9, ipsilateral hippocampus at M9, contralateral CCb from M6 to M9, contralateral CCs from M3 to M9, contralateral ab at M9, and contralateral cortex at M9, indicating that FA was reduced in TBI and/or rmTBI rats compared to shams. AD, RD, and MD values also gradually decreased or increased over time. The gradual changes of FA, AD, RD, and MD from M3 to M9 were generally associated with loss of white matter integrity after TBI. DTI enables measurement of the microstructural integrity of white matter in numerous experimental TBI studies of controlled cortical impact^6,49–54^. Simeone and colleagues^55^ have noted that in the subacute phase after TBI, DTI imaging is a predictive tool, indicating both the potential onset and anatomical localization of progressive lesions and providing insight into anticipated long-term neurological outcomes. These studies focused mainly on acute and subacute times after injury, and the consequences of slowing progressing secondary damage to tissue microstructure have not been evaluated. In 2015, Laitinen et al.^8^ investigated the detectability of secondary injury at a chronic time point using ex vivo and in vivo DTI in a rat model of TBI, lateral fluid percussion injury. Their results indicated decreases in FA but increases in both RD and MD in white matter areas, mainly associated with the loss of myelinated axons. Other investigations also showed that white matter atrophy was associated with elevated MD and reduced FA^46^. Consistent with the findings of Chary and colleagues^56^, the ic in TBI rats during the chronic phase exhibited a decrease in FA, a decrease in AD, an increase in RD, and an increase in MD, which can be attributed to microstructural changes, such as the loss of myelinated axons and/or the accumulation of iron deposits. However, their findings in the CCs region partially align with our results, showing a decrease in FA, an increase in RD, and an increase in MD. The discrepancy in the behavior of AD in the CCs region may stem from the proximity of CCs to the lesion. Both of their and our results are in part consistent with previous studies showing that decreased FA is noted in the white matter in a controlled cortical animal model of TBI^57,58^ and in TBI patients^59,60^. The in vivo DTI metrics can be biased by free water compartment at ROIs close to or located in lesion region with cerebral edema. Since free water component presents high diffusivity in all direction and low FA value, the elevated diffusivity and reduced FA can be observed depending on the amount of free water component within MR voxel^61^. This may be the reason why an increased AD in CCs is observed in our result but unchanged in ex vivo MRI experiment. To observe tissue-specific microstructure change, DTI can be combined with Fluid-Attenuated Inversion Recovery (FLAIR) technique to suppress the signal from CSF^62^or free water elimination to account for free water compartment^63^. Combining FLAIR with T2WI/T1WI images could potentially mitigate the effects of CSF on the images, allowing for a more comprehensive analysis of lesions. While our study has provided valuable insights into the domain of MRI-based lesion analysis, it is essential to acknowledge these limitations and use them as a basis for future research improvements to ensure the continued advancement of our understanding in this field.

Our present study describes and compares the behavioral and pathological consequences in a rat model of TBI and rmTBI administrated by LFP. Shams received only a craniectomy. Pathological changes in the brain microstructure at acute (3–7 days) and chronic (6–9 months) phases following TBI using MRI, neurobehavioral tests, and immunohistochemistry assays were performed from 7 days to 9 months after TBI. Our results revealed that TBI resulted in long-term cognitive, motor, and neurological deficits, whereas rmTBI resulted in more significant deficits in these parameters. Both in vivo DTI and LFB measurements also revealed that TBI causes noticeable microstructural tissue changes or thickness reduction in gray matter and white matter tracts of both hemispheres. Following TBI, the severity of neurobehavioral disorders was found to be significantly correlated with both the thickness of white matter and structural abnormalities at the nodes of Ranvier. Our data indicate that TBI might induce cognitive and motor deficits by causing bilateral white and gray matter injury. The loss of white matter was characterized by reduction in white matter thickness and downregulation of nodal and paranodal proteins at the nodes of Ranvier.

There are some limitations of the current animal model. It is well known that there is a notable difference in the anatomy and physiology of nonhuman mammals (in particular rodents) and human brains. Manganese-enhanced MRI scans revealed that LFP injury caused a 17% reduction in neurohypophyseal volume in the TBI group as compared to controls^64^, suggesting that TBI leads to pituitary atrophy and may serve as a prognostic biomarker for the post-TBI outcome. No single animal model will ever be able to replicate the complete spectrum of changes in human TBI. We used the stereotaxic apparatus according to the rat brain atlas with LFP to induce unilateral-parietal lobe injury, causing long-term cognitive and motor deficits and brain injury both ipsilateral and contralateral in rats. LFP is a diffuse model that delivers fluid pressure to the intact dura surface, creating a diffuse load to the brain. It should be emphasized that even when highly calibrated to ensure input equivalent, LFP still results in a spectrum of diffuse injuries, such as axonal swelling, rapid deformation, and a loss of connectivity, leading to tissue distortion and cavitation^65^. Our findings in the rat model suggest that TBI occurring within a short period can be catastrophic or fatal (evidenced by behavioral deficits and histopathological changes during long-term survival times). These data are consistent with findings in human patients who have experienced repeated brain concussions. Our data provide further insights into sports- and combat-related repeated concussions that should help clinical evaluations and control post-TBI outcomes. Furthermore, previous results observed the characteristics of white matter and gray matter in multiple sclerosis patients to identify changes in DTI-FA values following white matter injury^66^. They found that FA values were decreased in atrophic regions. The mechanism for white matter damage associated with brain atrophy may be that when the white matter myelin sheath is damaged, its neurotrophic effects on the gray matter are suppressed, which results in gray matter atrophy. Finally, it should be mentioned that in our study, survival differed between TBI and rmTBI (40% vs. 80%). The 60% that died introduces a selection bias of the type of TBI that produces myelin changes in the brain that correlate with histology. Whether a single mild TBI would produce changes would be of interest.

In our present study, only male mice were studied. Whether MRI-DTI applies to female mice will be necessary to understand. In fact, there is evidence of sex differences in outcomes after TBI in rodents and humans. For example, females had a lower rate of comorbidities and complications after TBI than males^67^. In MRI observations, female rats had reduced ipsilateral prefrontal cortex volume following rmTBI compared to female sham and mild TBI rats, while there were no differences among the male groups^68^. In contrast, only the male rats exhibited worse white matter integrity in the corpus callosum following rmTBI, not in female rats^68^. Furthermore, compared to male rats, female rats had superior vessel density and better neuromotor function following rmTBI^69^. These sexual dimorphisms in TBI responses may be in part due to endocrine-related differences, such as sex hormones^70^. Although the present study provides 9 months of MRI observations strengthened by complementary histological studies, we did not investigate the influences of sex differences in the pathogenesis of TBI or rmTBI. As such, future studies incorporating male and female cohorts are needed to examine how sex difference influences TBI-induced brain pathology and long-term functional outcomes to provide greater evidence for translation to the clinic.

## Conclusions

The current experiment explored the behavioral and histological effects of a single or repeated brain injury up to 9 months after experimental TBI in rats. At the histopathological level, we demonstrated the time course of white and gray matter atrophy, brain tissue degeneration, and blood–brain barrier disruption. MRI-DTI corroborated this finding. On the functional level, we demonstrated the development of motor and memory loss beginning 7 days after and progressing up to 9 months after TBI. Our data suggest that the white and gray matter loss (evaluated by MRI-DTI) can serve as noninvasive and reliable markers of cellular and behavioral level alterations in chronic TBI.

### Supplementary Information

Supplementary Information 1.

Supplementary Video 1.

## Data Availability

The data that support the findings of this study are available from the corresponding author upon reasonable request.

## Abbreviations

AD: Axial diffusivity
ab: Anterior commissure
BBB: Blood–brain barrier
Caspr: Contactin-associated protein
CCb: Body of corpus callosum
CCs: splenium of the corpus callosum
CE-T1WI: Contrast-enhanced T1-weighted imaging
cFA: Color-coded fractional anisotropy
DTI: Diffusion tensor imaging
FA: Fractional anisotropy
GM: Gray matter
ic: Internal capsule
LD: Laterodorsal thalamic nucleus
LFB: Luxol fast blue
LFP: Lateral fluid percussion
MD: Mean diffusivity
mNSS: Modified neurological severity score
MRI: Magnetic resonance imaging
TBI: Traumatic brain injury
RD: Radial diffusivity
ROI: Regions of interest
rmTBI: Repetitive mild traumatic brain injury
TTC: Triphenyl tetrazolium chloride
WM: White matter
VPL: Ventral posterolateral nucleus
VPM: Ventral posteromedial nucleus
