# Supplementary material for: White and gray matter integrity evaluated by MRI-DTI can serve as noninvasive and reliable indicators of structural and functional alterations in chronic neurotrauma
Source: Sci Rep. 2024 Mar 27;14:7244. doi: 10.1038/s41598-024-57706-7 (PMC10973494; doi:10.1038/s41598-024-57706-7)
Supplement: Supplementary file 1 — Supplementary Information 1. [file 41598_2024_57706_MOESM1_ESM.docx]

**Supplementary Data**

**White and gray matter integrity evaluated by MRI-DTI can serve as noninvasive and reliable indicators of structural and functional alterations in chronic neurotrauma**

**Lan-Wan Wang ^1,2^, Kuan-Hung Cho ^3^, Pi-Yu Chao ^4^, Li-Wei Kuo ^3,5^, Chia-Wen Chiang ^3^, Chien-Ming Chao ^6,7^, Mao-Tsun Lin ^4^, Ching-Ping Chang ^4,*^, Hung-Jung Lin ^8,9,*^, Chung-Ching Chio ^10,*^**

^1^Department of Pediatrics, Chi Mei Medical Center, Tainan 710, Taiwan

^2^Department of Biotechnology and Food Technology, Southern Taiwan University of Science and Technology, Tainan 710, Taiwan

^3^ Department of Electronic Engineering, National United University, Maioli City 360, Taiwan

^4^Department of Medical Research, Chi Mei Medical Center, Tainan 710, Taiwan

^5^Institute of Biomedical Engineering and Nanomedicine, National Health Research Institutes, Miaoli County 350, Taiwan.

^6^Institute of Medical Device and Imaging, National Taiwan University College of Medicine, Taipei 100, Taiwan

^7^Department of Intensive Care Medicine, Chi Mei Medical Center, Liouying, Tainan 73657, Taiwan.

^8^Department of Nursing, Min-Hwei College of Health Care Management, Tainan, Taiwan.

^9^Department of Emergency Medicine, Chi Mei Medical Center, Tainan 710, Taiwan

^10^School of Medicine, Taipei Medical University, Taipei 110, Taiwan

^11^Division of Neurosurgery, Department of Surgery, Chi Mei Medical Center, Tainan 710, Taiwan

**Correspondence to:**

**Ching-Ping Chang**, Ph.D., Department of Medical Research, Chi Mei Medical Center. Address: No. 901, Zhonghua Rd, Yongkang District, Tainan City 710, Taiwan. Phone: +886-6-2812811 ext 52657; fax: +886-6-2832639; e-mail: [jessica.cpchang@gmail.com](mailto:jessica.cpchang@gmail.com) or [a50831@mail.chimei.org.tw](mailto:a50831@mail.chimei.org.tw)

**Hung-Jung Lin**, MD., Department of Emergency Medicine, Chi Mei Medical Center. Address: No. 901, Zhonghua Rd, Yongkang District, Tainan City 710, Taiwan. Phone: +886-6-2812811 ext 52000; fax: +886-6-2832639; e-mail: [790001@mail.chimei.org.tw](mailto:790001@mail.chimei.org.tw)

**Chung-Ching Chio**, M.D., Division of Neurosurgery, Department of Surgery, Chi Mei Medical Center. Address: No. 901, Zhonghua Rd, Yongkang District, Tainan City 710, Taiwan. Phone: +886-6-2812811 ext 52000; fax: +886-6-2832639; e-mail: [chiocc@ms28.hinet.net](mailto:chiocc@ms28.hinet.net)

Total figure number: 7

**Legends for supplementary video 1:**

The time-dependent changes in DTI, T2WI, T1WI, and CE-T1WI whole-brain images from D3 to M9 from a sham rat, a TBI rat, and a rmTBI rat are shown in video format.


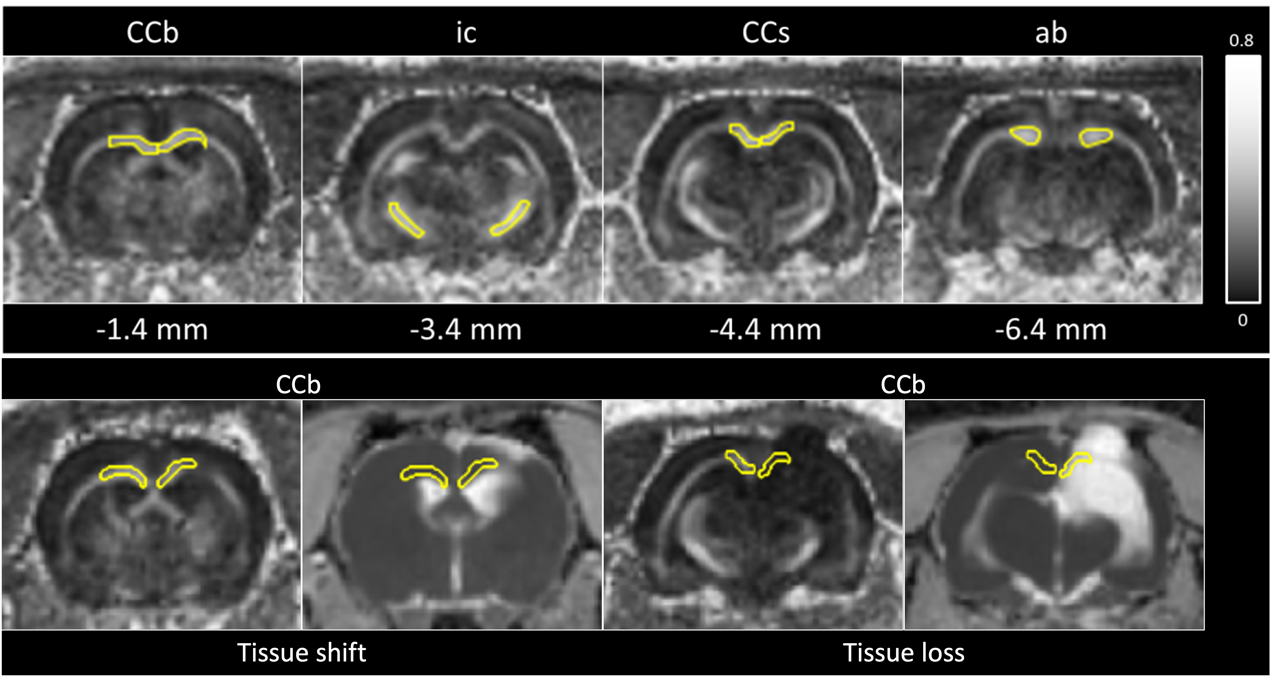


**Figure S1:** Top: Region of interests (ROIs) included in the DTI analysis. ROIs are outlined in a representative coronal FA map of a sham-operated animal. Four ROIs are outlined on body of corpus callosum (CCb), internal capsule (ic), splenium of corpus callosum (CCs) and angular bundle (ab) respectively. The contralateral hemisphere is on the left of image and the ipsilateral hemisphere is on the right of image. Gray scale indicates FA values between 0 (black) and 0.8 (white). Bottom: Examples of ROI selection for cases of shift of CCb (left) and loss of CCb (right). With the case of tissue loss, the ROI is outlined based on the rat brain atlas no matter the ROI is filled by CSF or not.


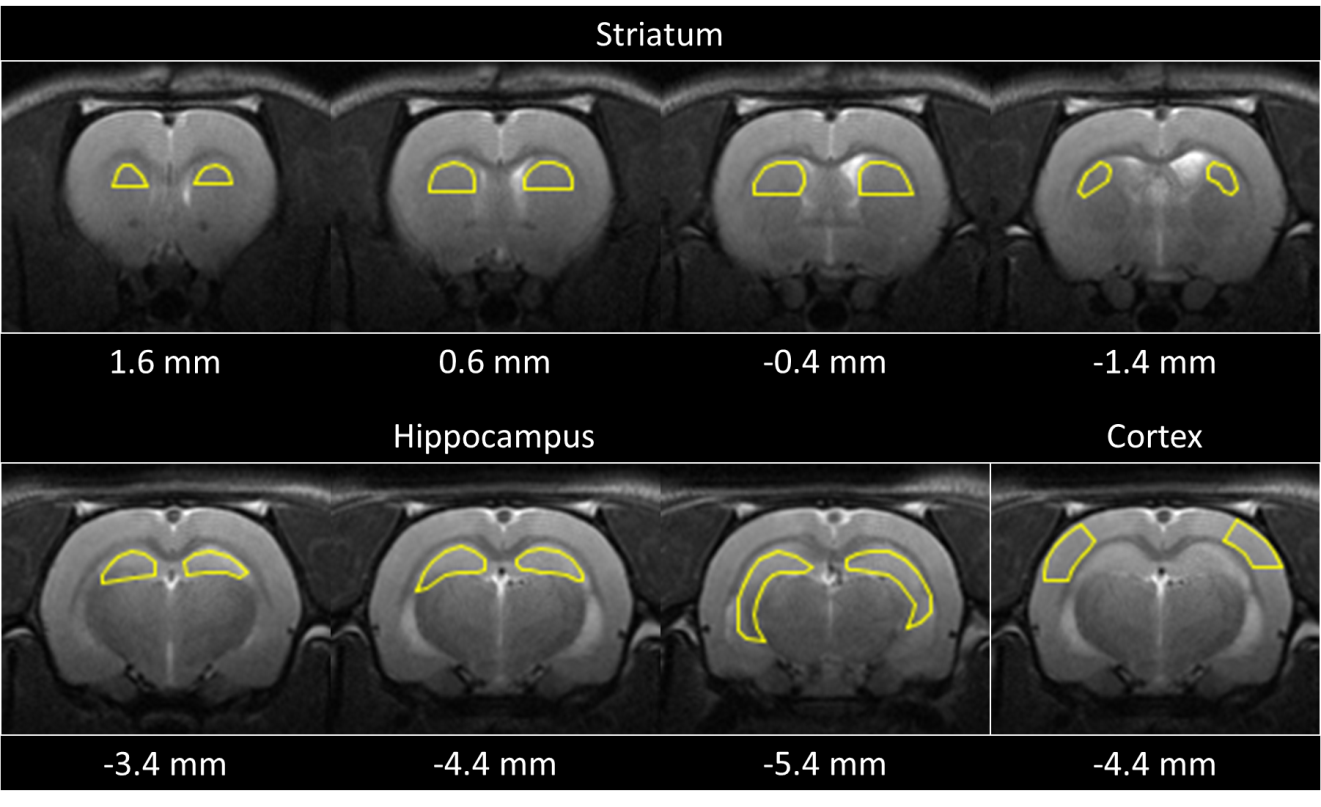


**Figure S2:** ROIs included in the T2WI analysis. ROIs are outlined in a representative coronal T2WI map of a sham-operated animal. Three ROIs are outline on striatum, hippocampus and cortex, respectively. The contralateral hemisphere is on the left of the image and the ipsilateral hemisphere is on the right of the image. All gray matter ROIs are selected based on the rat brain atlas no matter the ROI is filled by CSF or not.


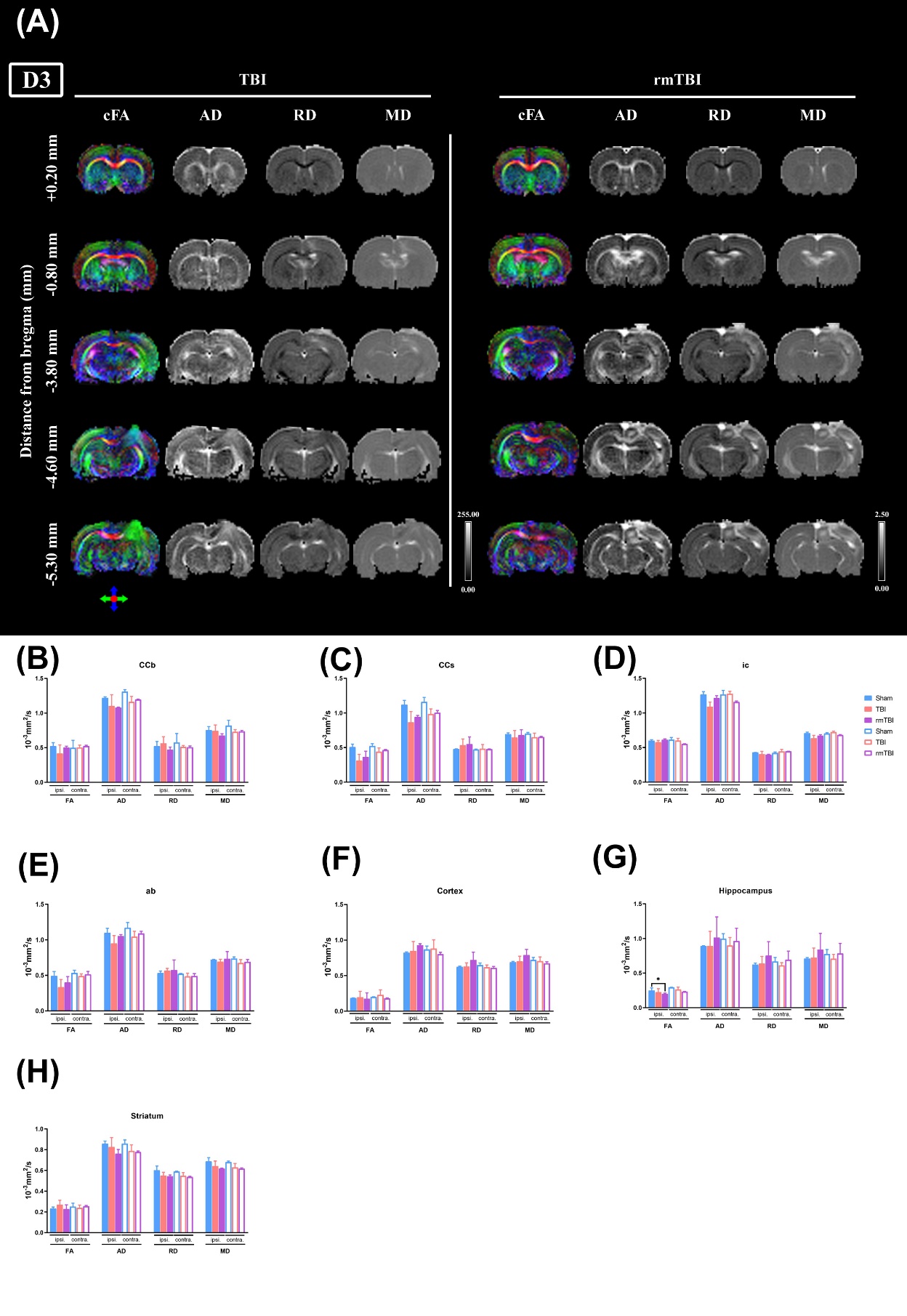


**Figure S3:** TBI causes alterations in DTI parameters at D3 after injury. **(A)** Coronal images of the diffusion parameter map (bregma +0.20 mm to -5.3 mm) were obtained from an TBI and an rmTBI rat on day 3 (D3) post-injury. (A) Coronal brain sections presented the colored fractional anisotropy (cFA), mean diffusivity (MD), radial diffusivity (RD), and axial diffusivity (AD) maps. The cFA brain map with direction encoded color maps were based on the extracted direction: red was for medial-lateral (x-axis), green was for rostral-caudal (y-axis), and blue was for dorsal-ventral (z-axis). The grayscale indicates FA values between 0 (black) and 1 (white) and AD, RD, and MD values between 0 (black) and 255 (white). The four indices FA, AD, RD, and MD for each brain structure are quantified at D3 of post-injury, including **(B)** CCb, **(C)** CCs, **(D)** ic, **(E)** ab, **(F)** cortex, **(G)** hippocampus, and **(H)** striatum. Blue bars represents Sham group (n = 2), orange bars TBI group (n = 4), and purple bars rmTBI group (n=2). The statistical analysis data are summarized in “**Table S2**”.

**
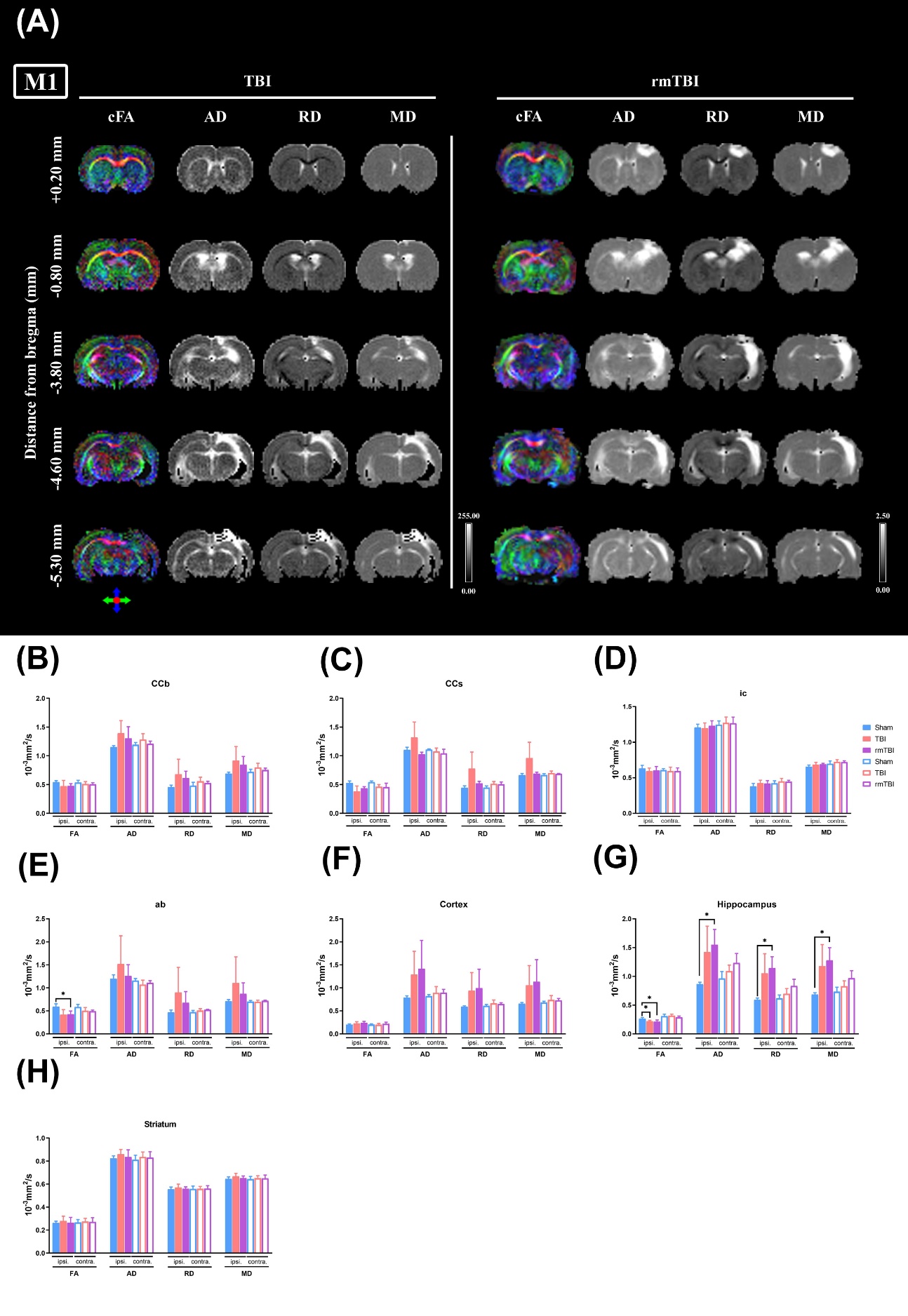
**

**Figure S4:** TBI causes alterations in DTI parameters at M1 after injury. **(A)** Coronal images of the diffusion parameter map (bregma +0.20 mm to -5.3 mm) were obtained from an TBI and an rmTBI rat on month 1 (M1) post-injury. Coronal brain sections presented the colored fractional anisotropy (cFA), mean diffusivity (MD), radial diffusivity (RD), and axial diffusivity (AD) maps. The cFA brain map with direction encoded color maps were based on the extracted direction: red was for medial-lateral (x-axis), green was for rostral-caudal (y-axis), and blue was for dorsal-ventral (z-axis). The grayscale indicates FA values between 0 (black) and 1 (white) and AD, RD, and MD values between 0 (black) and 255 (white). The four indices FA, AD, RD, and MD for each brain structure are quantified at M1 of post-injury, including **(B)** CCb, **(C)** CCs, **(D)** ic, **(E)** ab, **(F)** cortex, **(G)** hippocampus, and **(H)** striatum. Blue bars represents Sham group (n = 5), orange bars TBI group (n = 6), and purple bars rmTBI group (n = 4). The statistical analysis data were summarized in “**Table S3**”.


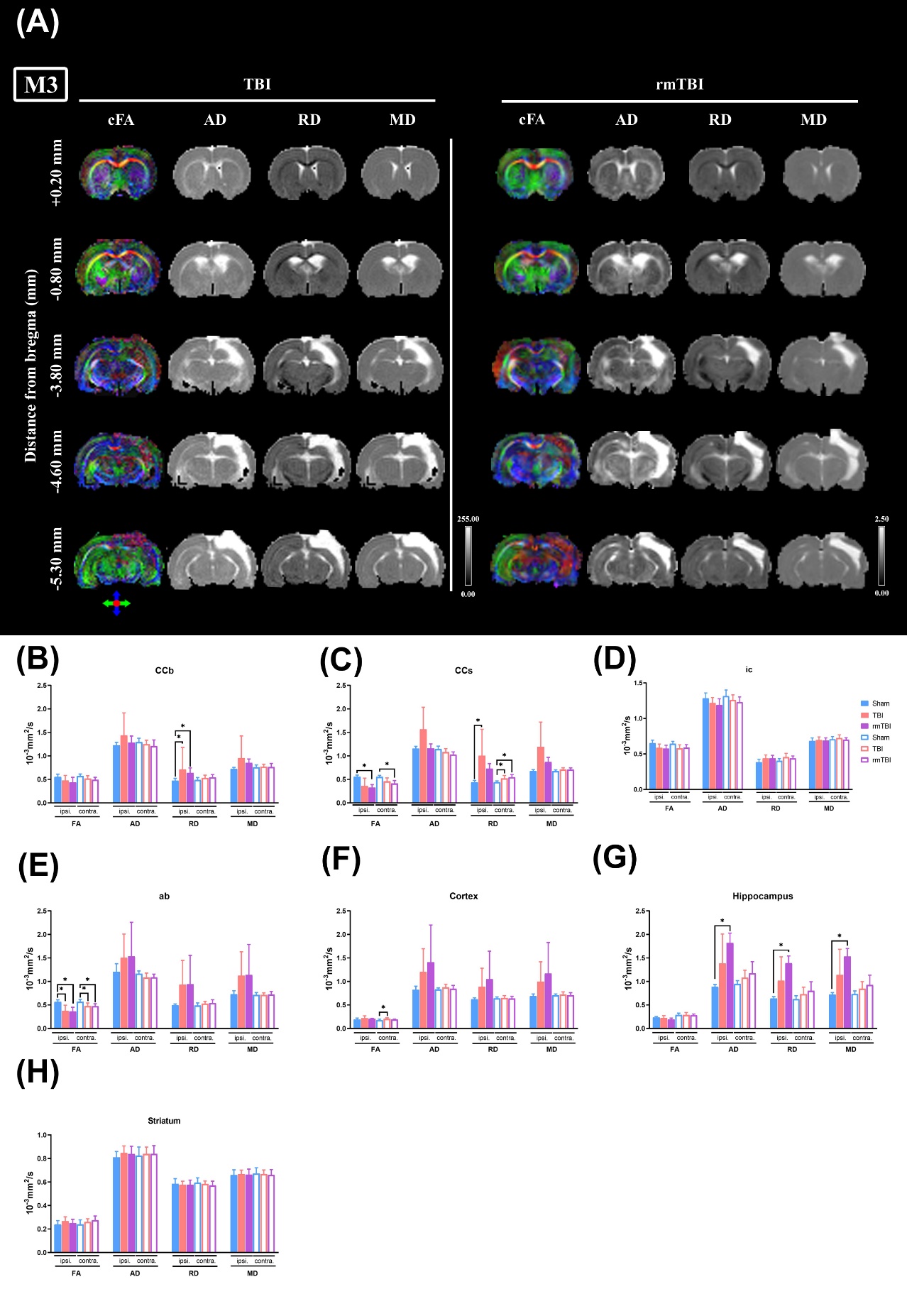


**Figure S5:** TBI causes alterations in DTI parameters at M3 after injury. **(A)** Coronal images of the diffusion parameter map (bregma +0.20 mm to -5.3 mm) were obtained from an mTBI and an rTBI rat on month 3 (M3) post-injury. Coronal brain sections presented the colored fractional anisotropy (cFA), mean diffusivity (MD), radial diffusivity (RD), and axial diffusivity (AD) maps. The cFA brain map with direction encoded color maps were based on the extracted direction: red was for medial-lateral (x-axis), green for rostral-caudal (y-axis), and blue for dorsal-ventral (z-axis). The grayscale indicates FA values between 0 (black) and 1 (white) and AD, RD, and MD values between 0 (black) and 255 (white). The four indices FA, AD, RD, and MD for each brain structure are analyzed at M3 of post-injury, including **(B)** CCb, **(C)** CCs, **(D)** ic, **(E)** ab, **(F)** cortex, **(G)** hippocampus, and **(H)** striatum. Blue bars represents Sham group (n = 6), orange bars TBI group (n = 5), and purple bars rmTBI group (n = 6). The statistical analysis data were summarized in in “**Table S4**”.


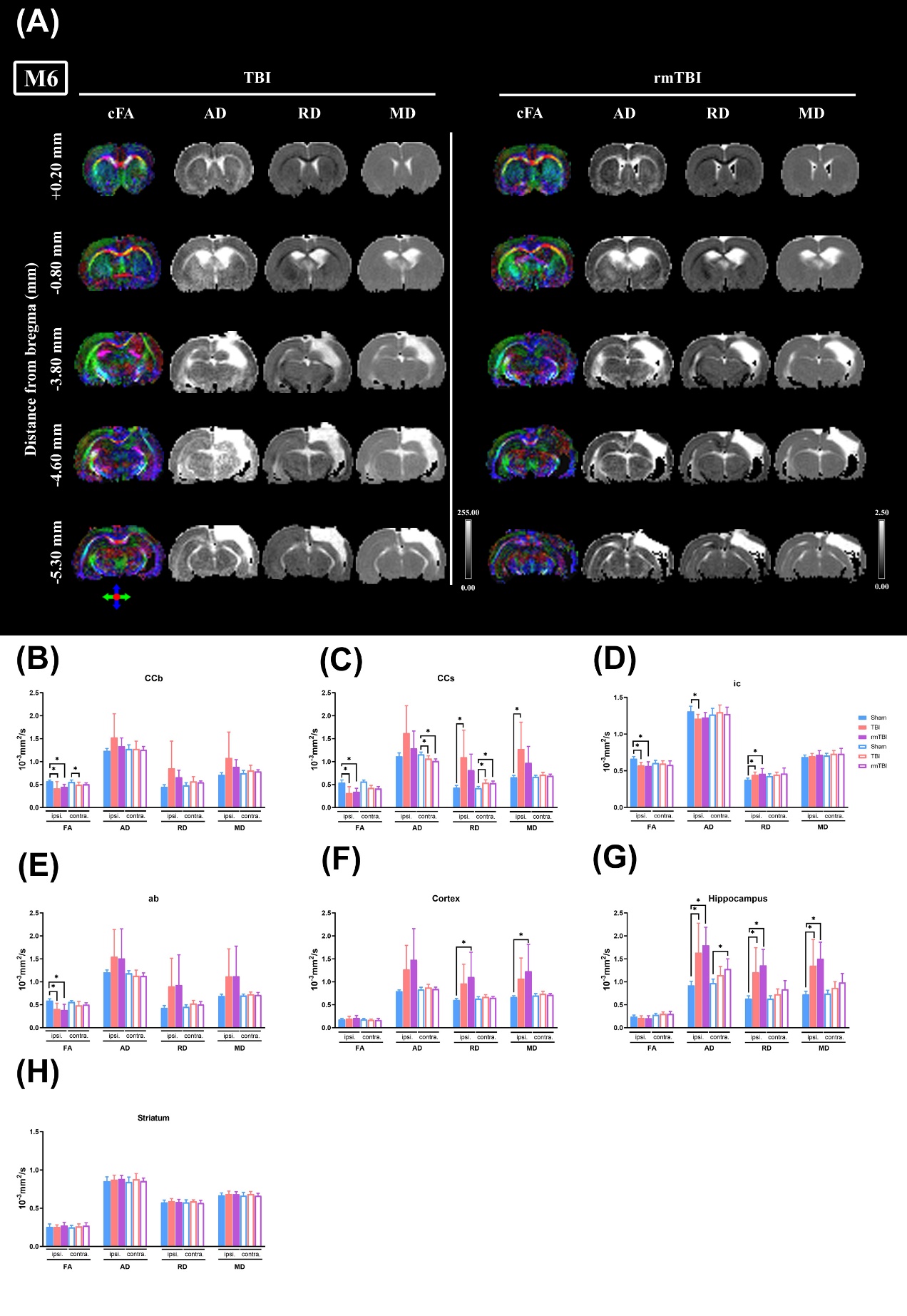


**Figure S6:** TBI causes alterations in DTI parameters at M6 after injury. **(A)** Coronal images of the diffusion parameter map (bregma +0.20 mm to -5.3 mm) were obtained from an mTBI and an rTBI rat on month 6 (M6) post-injury. Coronal brain sections presented the colored fractional anisotropy (cFA), mean diffusivity (MD), radial diffusivity (RD), and axial diffusivity (AD) maps. The cFA brain map with direction encoded color maps were based on the extracted direction: red was for medial-lateral (x-axis), green for rostral-caudal (y-axis), and blue for dorsal-ventral (z-axis). The grayscale indicates FA values between 0 (black) and 1 (white) and AD, RD, and MD values between 0 (black) and 255 (white). The four indices FA, AD, RD, and MD for each brain structure are analyzed at M6 of post-injury, including **(B)** CCb, **(C)** CCs, **(D)** ic, **(E)** ab, **(F)** cortex, **(G)** hippocampus, and **(H)** striatum. Blue bars represents Sham group (n = 9), orange bars TBI group (n = 10), and purple bars rmTBI group (n = 8). The statistical analysis data were summarized in “**Table S5**”.

**
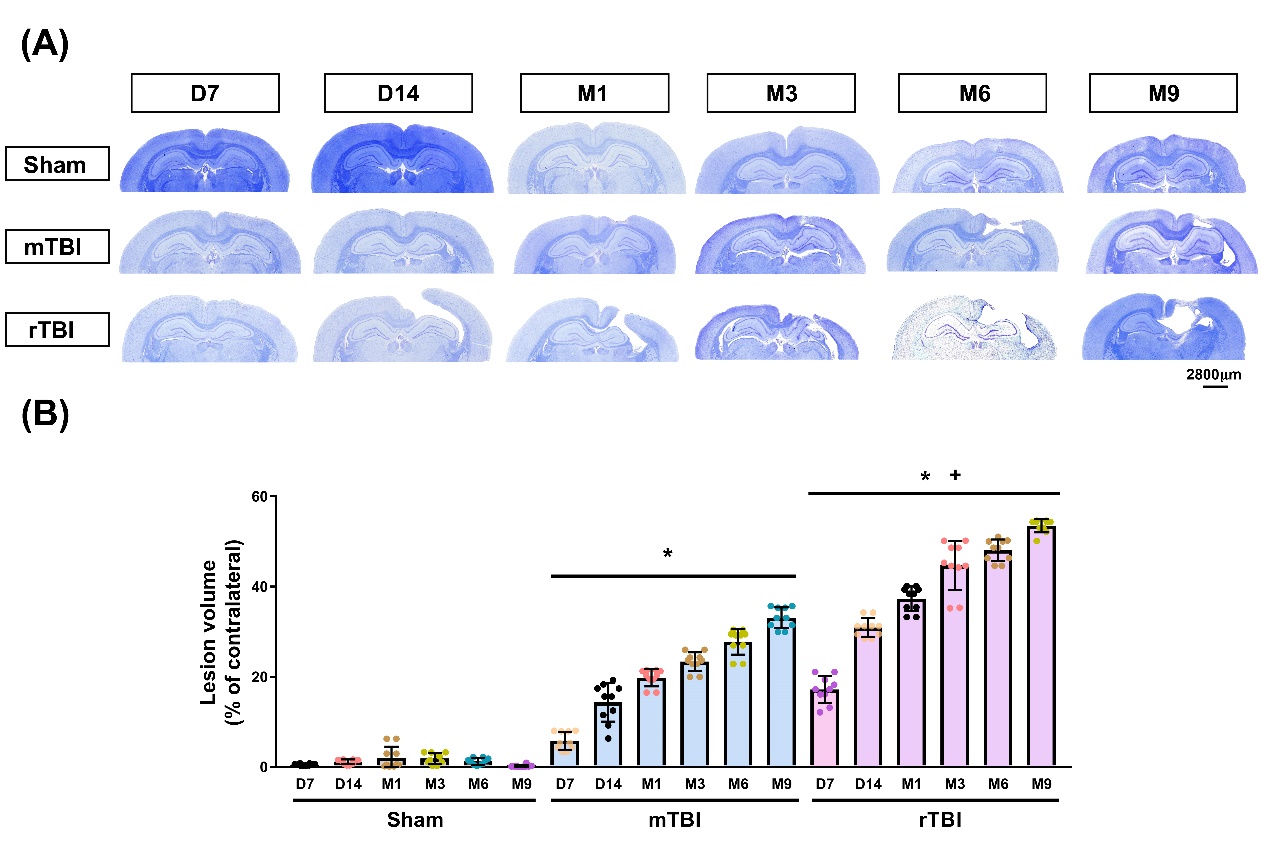
**

**Figure S7: TBI causes a reduction of both brain cavity and white matter thickness.** Luxol Fast Blue (LFB) and cresyl violet (or Nissl) staining show the brain lesion's temporal progression. LFB stain is used to identify myelin. Cresyl violet is used to stain neuronal cell bodies, processes, and Nissl bodies. (**A**) The representative of LFB with Nissl staining from day 7 (D7) to months 9 (M9) post-sham operation, post-TBI, and post-rmTBI. (**B**) Quantification of brain cavity volume (lesion volume) in different severities of TBI. Data are presented as mean±SD (*n*=10). * *P* <0.05, compared with the Sham group. + *P* <0.05, compared with mTBI group.

**Table S1: All figures' statistical data**

| **Figure No.** | **Statistics data** | | |
| --- | --- | --- | --- |
| 2A | **Survival** | **Chi^2^ / df** | **P value** |
|  | Log-rank (Mantel-Cox) test (recommended) | 25.19 / 2 | <0.0001 |
|  | Logrank test for trend (recommended) | 2.403 / 1 | 0.1211 |
|  | Gehan-Breslow-Wilcoxon test | 22.25 / 2 | <0.0001 |
| 2C | **Contusion volume** | **F (DFn, DFd)** | **P value** |
|  | Interaction (Group × Time) | F (10, 107) = 40.48 | P<0.0001 |
|  | Time Factor | F (5, 107) = 86.10 | P<0.0001 |
|  | Group Factor | F (2, 107) = 317.6 | P<0.0001 |
| 3A | **Y maze** | **F (DFn, DFd)** | **P value** |
|  | Interaction (Group × Time) | F (12, 319) = 10.83 | P<0.0001 |
|  | Time Factor | F (6, 319) = 20.38 | P<0.0001 |
|  | Group Factor | F (2, 319) = 170.1 | P<0.0001 |
| 3B | **Radial maze: Retention time** | **F (DFn, DFd)** | **P value** |
|  | Interaction (Group × Time) | F (12, 319) = 8.6831 | P<0.0001 |
|  | Time Factor | F (6, 319) = 34.53 | P<0.0001 |
|  | Group Factor | F (2, 319) = 8.683 | P<0.0001 |
| 3C | **Radial maze: working memory** | **F (DFn, DFd)** | **P value** |
|  | Interaction (Group × Time) | F (12, 319) = 3.211 | P=0.0012 |
|  | Time Factor | F (6, 319) = 9.497 | P<0.0001 |
|  | Group Factor | F (2, 319) = 57.42 | P<0.0001 |
| 3D | **Radial maze: reference memory** | **F (DFn, DFd)** | **P value** |
|  | Interaction (Group × Time) | F (12, 319) = 4.155 | P<0.0001 |
|  | Time Factor | F (6, 319) = 55.29 | P<0.0001 |
|  | Group Factor | F (2, 319) = 97.26 | P<0.0001 |
| 3E | **Passive avoidance: latency** | **F (DFn, DFd)** | **P value** |
|  | Interaction (Group × Time) | F (12, 319) = 5.687 | P<0.0001 |
|  | Time Factor | F (6, 319) = 30.64 | P<0.0001 |
|  | Group Factor | F (2, 266) = 26.89 | P<0.0001 |
| 3F | **Passive avoidance: number of error** | **F (DFn, DFd)** | **P value** |
|  | Interaction (Group × Time) | F (12, 319) = 5.133 | P<0.0001 |
|  | Time Factor | F (6, 319) = 22.58 | P<0.0001 |
|  | Group Factor | F (2, 319) = 11.29 | P<0.0001 |
| 3G | **Rotarod: Mean velocity** | **F (DFn, DFd)** | **P value** |
|  | Interaction (Group × Time) | F (12, 319) = 48.9 | P<0.0001 |
|  | Time Factor | F (6, 319) = 78.11 | P<0.0001 |
|  | Group Factor | F (2, 319) = 612 | P<0.0001 |
| 3H | **Rotarod: latency** | **F (DFn, DFd)** | **P value** |
|  | Interaction (Group × Time) | F (12, 319) = 52.93 | P<0.0001 |
|  | Time Factor | F (6, 319) = 77.64 | P<0.0001 |
|  | Group Factor | F (2, 319) = 705.9 | P<0.0001 |
| 3I | **Inclined plane** | **F (DFn, DFd)** | **P value** |
|  | Interaction (Group × Time) | F (12, 319) = 69.54 | P<0.0001 |
|  | Time Factor | F (6, 319) = 282.3 | P<0.0001 |
|  | Group Factor | F (2, 319) = 796.6 | P<0.0001 |
| 3J | **mNSS** | **F (DFn, DFd)** | **P value** |
|  | Interaction (Group × Time) | F (12, 319) = 94.69 | P<0.0001 |
|  | Time Factor | F (6, 319) = 541.5 | P<0.0001 |
|  | Group Factor | F (2, 319) = 1508 | P<0.0001 |
| 4B | **HE stain-Hippocampus-CA1** | **F (DFn, DFd)** | **P value** |
|  | Interaction (Group × Time) | F (10, 135) = 4.751 | P<0.0001 |
|  | Time Factor | F (5, 135) = 25.59 | P<0.0001 |
|  | Group Factor | F (2, 135) = 289.9 | P<0.0001 |
| 4C | **HE stain-Hippocampus-CA2** | **F (DFn, DFd)** | **P value** |
|  | Interaction (Group × Time) | F (10, 135) = 2.783 | P=0.1853 |
|  | Time Factor | F (5, 135) = 19.25 | P<0.0001 |
|  | Group Factor | F (2, 135) = 374.4 | P<0.0001 |
| 4D | **HE stain-Hippocampus-CA3** | **F (DFn, DFd)** | **P value** |
|  | Interaction (Group × Time) | F (10, 135) = 3.510 | P=0.0004 |
|  | Time Factor | F (5, 135) = 14.04 | P<0.0001 |
|  | Group Factor | F (2, 135) = 308.6 | P<0.0001 |
| 4E | **HE stain-Hippocampus-DG** | **F (DFn, DFd)** | **P value** |
|  | Interaction (Group × Time) | F (10, 135) = 1.353 | P=0.1921 |
|  | Time Factor | F (5, 135) = 2.289 | P<0.0001 |
|  | Group Factor | F (2, 135) = 139.7 | P<0.0001 |
| 4F | **HE stain-cortex** | **F (DFn, DFd)** | **P value** |
|  | Interaction (Group × Time) | F (10, 135) = 44.152 | P<0.0001 |
|  | Time Factor | F (5, 135) = 22.33 | P<0.0001 |
|  | Group Factor | F (2, 135) = 236.5 | P<0.0001 |
| 4G | **HE stain-Hypothalamus** | **F (DFn, DFd)** | **P value** |
|  | Interaction (Group × Time) | F (10, 135) = 1.72 | P=0.5973 |
|  | Time Factor | F (5, 135) = 1.0443 | P=0.5206 |
|  | Group Factor | F (2, 135) = 2.892 | P=0.0094 |
| 4H | **HE stain-striatum** | **F (DFn, DFd)** | **P value** |
|  | Interaction (Group × Time) | F (10, 135) = 2.699 | P=0.0053 |
|  | Time Factor | F (5, 135) = 2.621 | P=0.0026 |
|  | Group Factor | F (2, 135) = 7.595 | P=0.028 |
| 5B | **Contusion volume: TTC with MRI-T2WI** | **F (DFn, DFd)** | **P value** |
|  | Interaction (Group × Time) | F (20, 93) = 0.2927 | P=0.9986 |
|  | Time Factor | F (4, 93) = 0.6377 | P=0.6369 |
|  | Group Factor | F (5, 93) = 67.35 | P<0.0001 |
| 6B | **MRI-T1WI** | **F (DFn, DFd)** | **P value** |
|  | Interaction (Group × Time) | F (8, 53) = 1.085 | P=0.3877 |
|  | Time Factor | F (4, 53) = 8.742 | P<0.0001 |
|  | Group Factor | F (2, 53) = 2.852 | P=0.0759 |
| 8B | **LFB stain-Hippocampus** | **F (DFn, DFd)** | **P value** |
|  | Interaction (Group × Time) | F (10, 135) = 129.7 | P<0.0001 |
|  | Time Factor | F (5, 135) = 407.8 | P<0.0001 |
|  | Group Factor | F (2, 135) = 5631 | P<0.0001 |
| 9B | **LFB stain-CCb-Ipsilateral** | **F (DFn, DFd)** | **P value** |
|  | Interaction (Group × Time) | F (10, 135) = 7.282 | P<0.0001 |
|  | Time Factor | F (5, 135) = 58.36 | P<0.0001 |
|  | Group Factor | F (2, 135) = 228.7 | P<0.0001 |
| 9C | **LFB stain-CCb-Contralateral** | **F (DFn, DFd)** | **P value** |
|  | Interaction (Group × Time) | F (10, 135) = 4.534 | P<0.0001 |
|  | Time Factor | F (5, 135) = 1.534 | P<0.0001 |
|  | Group Factor | F (2, 135) = 222.7 | P<0.0001 |
| 9D | **LFB stain-ab-Ipsilateral** | **F (DFn, DFd)** | **P value** |
|  | Interaction (Group × Time) | F (10, 135) = 3.574 | P<0.0001 |
|  | Time Factor | F (5, 135) = 10.08 | P<0.0001 |
|  | Group Factor | F (2, 135) = 399.6 | P<0.0001 |
| 9E | **LFB stain-ab-Contralateral** | **F (DFn, DFd)** | **P value** |
|  | Interaction (Group × Time) | F (10, 135) = 3.5744 | P=0.0742 |
|  | Time Factor | F (5, 135) = 6.237 | P=0.0003 |
|  | Column Factor | F (2, 135) = 51.05 | P<0.0001 |
| 10B | **Ankyrin G+Caspr-CCb** | **F (DFn, DFd)** | **P value** |
|  | Interaction (Group × Time) | F (10, 135) = 10.25 | P<0.0001 |
|  | Time Factor | F (5, 135) = 28.43 | P<0.0001 |
|  | Group Factor | F (2, 135) = 735.1 | P<0.0001 |
| 10C | **Ankyrin G+Caspr-LD** | **F (DFn, DFd)** | **P value** |
|  | Interaction (Group × Time) | F (10, 135) = 0.5735 | P=0.8324 |
|  | Time Factor | F (5, 135) = 0.9880 | P=0.4285 |
|  | Group Factor | F (2, 135) = 0.4572 | P=0.5273 |
| 10D | **Ankyrin G+Caspr-VPL-VPM** | **F (DFn, DFd)** | **P value** |
|  | Interaction (Group × Time) | F (10, 135) = 1.344 | P=0.2674 |
|  | Time Factor | F (5, 135) = 1.301 | P=0.1493 |
|  | Column Factor | F (2, 135) = 0.1929 | P=0.2134 |
| 10E | **Ankyrin G+Caspr-ic** | **F (DFn, DFd)** | **P value** |
|  | Interaction (Group × Time) | F (10, 135) = 1.299 | P=0.2402 |
|  | Time Factor | F (5, 135) = 1.202 | P=0.3128 |
|  | Group Factor | F (2, 135) = 0.6889 | P=0.5114 |
| 10F | **Ankyrin G+Caspr-ab** | **F (DFn, DFd)** | **P value** |
|  | Interaction (Group × Time) | F (10, 135) = 8.366 | P<0.0001 |
|  | Time Factor | F (5, 135) = 4.139 | P=0.0016 |
|  | Group Factor | F (2, 135) = 390.5 | P<0.0001 |

**TABLE S2:** Fractional anisotropy (FA) and axial diffusivity (AD), radial diffusivity (RD), mean diffusivity (MD) from ipsilateral and contralateral hemisphere in sham control rats (n=2) and rats post-mTBI (n=4) or post-rTBI at **day3** (n=2) obtained using *in vivo* DTI.

|  |  | **FA** | | | | |  | **AD (µm^2^/ms)** | | | | |  | **RD (µm^2^/ms)** | | | | |  | **MD (µm^2^/ms)** | | | | |
| --- | --- | --- | --- | --- | --- | --- | --- | --- | --- | --- | --- | --- | --- | --- | --- | --- | --- | --- | --- | --- | --- | --- | --- | --- |
|  |  | **Sham** | **mTBI** | **rTBI** | **F (DFn, DFd)** | **P value** |  | **Sham** | **mTBI** | **rTBI** | **F(DFn, DFd)** | **P value** |  | **Sham** | **mTBI** | **rTBI** | **F (DFn, DFd)** | **P value** |  | **Sham** | **mTBI** | **rTBI** | **F(DFn, DFd)** | **P value** |
| **Ipsilateral** | ***WM:*** |  |  |  |  |  |  |  |  |  |  |  |  |  |  |  |  |  |  |  |  |  |  |  |
|  | CCb | 0.52±0.05 | 0.42±0.12 | 0.50±0.02 | F (2,5) = 1.835 | 0.3527 |  | 1.22±0.02 | 1.10±0.16 | 1.08±0.01 | F (2,5) = 16.17 | 0.0582 |  | 0.52±0.07 | 0.56±0.10 | 0.47±0.04 | F (2,5) = 1.588 | 0.3864 |  | 0.75±0.05 | 0.74±0.09 | 0.67±0.03 | F (2,5) = 4.619 | 0.178 |
|  | CCs | 0.51±0.04 | 0.31±0.09 | 0.36±0.08 | F (2,5) = 24.55 | 0.0391 |  | 1.12±0.06 | 0.86±0.16 | 0.94±0.02 | F (2,5) = 3.109 | 0.2434 |  | 0.48±0.00 | 0.53±0.09 | 0.55±0.10 | F (2,5) = 0.3966 | 0.716 |  | 0.69±0.02 | 0.64±0.10 | 0.68±0.08 | F(2,5)=1.201 | 0.375 |
|  | ic | 0.60±0.01 | 0.58±0.03 | 0.61±0.01 | F (2,5) = 1.859 | 0.3497 |  | 1.27±0.04 | 1.09±0.07 | 1.22±0.03 | F (2,5) = 7.308 | 0.0328 |  | 0.43±0.00 | 0.40±0.04 | 0.40±0.01 | F (2,5) = 35.50 | 0.0274 |  | 0.71±0.01 | 0.63±0.04 | 0.67±0.02 | F (2,5) = 5.677 | 0.1498 |
|  | ab | 0.49±0.06 | 0.33±0.11 | 0.40±0.08 | F(2,5)=1.914 | 0.2415 |  | 1.10±0.07 | 0.95±0.11 | 1.05±0.02 | F (2,5) = 1.986 | 0.3349 |  | 0.53±0.03 | 0.56±0.03 | 0.57±0.14 | F (2,5) = 0.2100 | 0.8264 |  | 0.72±0.00 | 0.69±0.03 | 0.73±0.10 | F (2,5) = 0.4847 | 0.6735 |
|  | ***GM:*** |  |  |  |  |  |  |  |  |  |  |  |  |  |  |  |  |  |  |  |  |  |  |  |
|  | Cortex | 0.18±0.00 | 0.20±0.08 | 0.17±0.08 | F (2,5) = 0.05497 | 0.9479 |  | 0.82±0.01 | 0.85±0.13 | 0.93±0.02 | F (2,5) = 8.218 | 0.1085 |  | 0.62±0.01 | 0.63±0.05 | 0.72±0.11 | F (2,5) = 2.411 | 0.2932 |  | 0.69±0.01 | 0.70±0.07 | 0.79±0.08 | F (2,5) = 7.931 | 0.112 |
|  | Hippo. | 0.21±0.01 | 0.23±0.07 | 0.24±0.05 | F (2,5) = 1.834 | 0.3529 |  | 0.88±0.00 | 0.94±0.15 | 0.99±0.15 | F (2,5) = 0.2511 | 0.7993 |  | 0.64±0.00 | 0.67±0.07 | 0.69±0.04 | F (2,5) = 1.012 | 0.4971 |  | 0.72±0.00 | 0.76±0.09 | 0.79±0.07 | F (2,5 ) = 0.6238 | 0.6158 |
|  | Stria. | 0.22±0.01 | 0.22±0.03 | 0.24±0.03 | F (2,2) = 1.039 | 0.4904 |  | 0.83±0.00 | 0.77±0.09 | 0.76±0.02 | F (2,5) = 6.971 | 0.1255 |  | 0.61±0.02 | 0.57±0.05 | 0.55±0.01 | F (2,5) = 2.353 | 0.2983 |  | 0.68±0.02 | 0.64±0.06 | 0.62±0.00 | F (2,5) = 13.70 | 0.068 |
|  |  |  |  |  |  |  |  |  |  |  |  |  |  |  |  |  |  |  |  |  |  |  |  |  |
| **Contralateral** | ***WM:*** |  |  |  |  |  |  |  |  |  |  |  |  |  |  |  |  |  |  |  |  |  |  |  |
|  | CCb | 0.50±0.11 | 0.50±0.03 | 0.52±0.01 | F (2,5) = 0.1262 | 0.8841 |  | 1.31±0.03 | 1.16±0.08 | 1.20±0.01 | F (2,5) = 44.65 | 0.0219 |  | 0.58±0.13 | 0.51±0.02 | 0.51±0.02 | F (2,5) = 0.8650 | 0.5362 |  | 0.82±0.08 | 0.73±0.03 | 0.74±0.01 | F (2,5) = 0.3250 | 0.1247 |
|  | CCs | 0.52±0.03 | 0.44±0.05 | 0.46±0.01 | F (2,5) = 2.381 | 0.2958 |  | 1.16±0.06 | 0.98±0.07 | 1.00±0.03 | F (2,5) = 5.355 | 0.1574 |  | 0.47±0.01 | 0.48±0.06 | 0.48±0.00 | F (2,5) = 5.788 | 0.1473 |  | 0.70±0.02 | 0.65±0.06 | 0.65±0.01 | F (2,5) = 25.03 | 0.0384 |
|  | ic | 0.62±0.03 | 0.60±0.03 | 0.55±0.00 | F (2,5) = 3.655 | 0.2148 |  | 1.27±0.06 | 1.28±0.03 | 1.16±0.01 | F (2,5) = 8.794 | 0.1021 |  | 0.42±0.01 | 0.45±0.03 | 0.45±0.00 | F (2,5) = 1.222 | 0.4501 |  | 0.70±0.01 | 0.72±0.01 | 0.68±0.00 | F (2,5) = 6.887 | 0.1268 |
|  | ab | 0.53±0.04 | 0.49±0.03 | 0.52±0.04 | F(2,5)=  1.109 | 0.4741 |  | 1.17±0.07 | 1.05±0.08 | 1.09±0.03 | F (2,5) = 18.75 | 0.0506 |  | 0.52±0.00 | 0.49±0.05 | 0.49±0.3 | F (2,5) = 4.101 | 0.196 |  | 0.74±0.02 | 0.67±0.05 | 0.69±0.03 | F (2,5) = 34.83 | 0.0279 |
|  | ***GM:*** |  |  |  |  |  |  |  |  |  |  |  |  |  |  |  |  |  |  |  |  |  |  |  |
|  | Cortex | 0.20±0.00 | 0.23±0.07 | 0.18±0.01 | F (2,5) = 6.158 | 0.1397 |  | 0.87±0.05 | 0.88±0.12 | 0.80±0.03 | F (2,5) = 16.17 | 0.0583 |  | 0.65±0.03 | 0.62±0.03 | 0.61±0.02 | F (2,5) = 5.201 | 0.1613 |  | 0.72±0.04 | 0.71±0.06 | 0.67±0.02 | F (2,5) = 7.865 | 0.1128 |
|  | Hippo. | 0.25±0.01 | 0.22±0.04 | 0.24±0.01 | F(2,5)= 44.73 | 0.0219 |  | 0.95±0.02 | 0.87±0.10 | 0.93±0.11 | F (2,5) = 0.4618 | 0.6841 |  | 0.66±0.01 | 0.62±0.03 | 0.64±0.06 | F (2,5) = 1.051 | 0.4875 |  | 0.76±0.02 | 0.71±0.05 | 0.74±0.08 | F (2,5) = 0.7100 | 0.5848 |
|  | Stria. | 0.25±0.02 | 0.24±0.03 | 0.24±0.02 | F (2,5) = 0.3227 | 0.7382 |  | 0.85±0.02 | 0.79±0.05 | 0.74±0.01 | F (2,5)= 24.22 | 0.0396 |  | 0.60±0.00 | 0.56±0.03 | 0.54±0.01 | F (2,5) = 104.6 | 0.0095 |  | 0.68±0.01 | 0.63±0.03 | 0.61±0.00 | F (2,5) = 112.5 | 0.0088 |

Abbreviations: CCb, body of the corpus callosum; CCs, splenium of the corpus callosum; ic, internal capsule; ab, anterior commissure; Hippo, hippocampus; Stria.: striatum; WM, white matter; GM, gray matter.

**TABLE S3:** Fractional anisotropy (FA) and axial diffusivity (AD), radial diffusivity (RD), mean diffusivity (MD) from ipsilateral and contralateral hemisphere in sham control (n=5) and rats after mTBI (n=6) or rTBI at **Month 1** (n=4) obtained using *in vivo* DTI.

|  |  | **FA** | | | | |  | **AD (µm^2^/ms)** | | | | |  | **RD (µm^2^/ms)** | | | | |  | **MD (µm^2^/ms)** | | | | |
| --- | --- | --- | --- | --- | --- | --- | --- | --- | --- | --- | --- | --- | --- | --- | --- | --- | --- | --- | --- | --- | --- | --- | --- | --- |
|  |  | Sham | mTBI | rTBI | F (DFn, DFd) | P value |  | Sham | mTBI | rTBI | F (DFn, DFd) | P value |  | Sham | mTBI | rTBI | F (DFn, DFd) | P value |  | Sham | mTBI | rTBI | F (DFn, DFd) | P value |
| **Ipsilateral** | ***WM:*** |  |  |  |  |  |  |  |  |  |  |  |  |  |  |  |  |  |  |  |  |  |  |  |
|  | CCb | 0.54±0.03 | 0.47±0.01 | 0.48±0.04 | F (2,12) = 1.673 | 0.2286 |  | 1.15±0.02 | 1.40±0.22 | 1.30±0.20 | F (2,12) = 2.671 | 0.1098 |  | 0.46±0.03 | 0.68±0.26 | 0.61±0.12 | F(2,12)=2.103 | 0.1648 |  | 0.69±0.03 | 0.92±0.24 | 0.84±0.14 | F (2,12) = 2.389 | 0.1339 |
|  | CCs | 0.53±0.03 | 0.38±0.09 | 0.43±0.03 | F (2,12) = 6.902 | 0.0101 |  | 1.10±0.05 | 1.32±0.27 | 1.03±0.03 | F (2,12) = 3.779 | 0.0533 |  | 0.44±0.03 | 0.78±0.29 | 0.52±0.03 | F (2,12) = 4.838 | 0.0288 |  | 0.66±0.03 | 0.96±0.28 | 0.69±0.02 | F (2,12) = 4.440 | 0.036 |
|  | ic | 0.63±0.04 | 0.59±0.05 | 0.60±0.05 | F (2,12) = 1.035 | 0.4039 |  | 1.21±0.05 | 1.20±0.07 | 1.23±0.07 | F (2,12) = 0.2970 | 0.7484 |  | 0.38±0.04 | 0.43±0.04 | 0.42±0.04 | F (2,12) = 1.992 | 0.2066 |  | 0.66±0.02 | 0.68±0.03 | 0.69±0.01 | F (2,12) = 2.773 | 0.1298 |
|  | ab | 0.59±0.06 | 0.42±0.11 | 0.43±0.07 | F(2,12)=  7.888 | 0.0161 |  | 1.20±0.09 | 1.52±0.61 | 1.26±0.24 | F (2,12) = 1.323 | 0.3255 |  | 0.47±0.05 | 0.90±0.55 | 0.68±0.24 | F (2,12) = 2.958 | 0.1172 |  | 0.71±0.04 | 1.11±0.57 | 0.87±0.24 | F (2,12) = 2.289 | 0.1718 |
|  | ***GM:*** |  |  |  |  |  |  |  |  |  |  |  |  |  |  |  |  |  |  |  |  |  |  |  |
|  | Cortex | 0.20±0.02 | 0.23±0.04 | 0.24±0.03 | F (2,12) = 2.017 | 0.2034 |  | 0.79±0.04 | 1.29±0.51 | 1.41±0.62 | F (2,12) = 2.582 | 0.1168 |  | 0.59±0.02 | 0.94±0.39 | 0.99±0.41 | F (2,12) =2.168 | 0.1571 |  | 0.66±0.03 | 1.05±0.43 | 1.33±0.48 | F (2,12) = 2.344 | 0.1383 |
|  | Hippo. | 0.25±0.03 | 0.22±0.01 | 0.23±0.02 | F (2,12) = 11.00 | 0.0069 |  | 0.87±0.04 | 1.23±0.33 | 1.39±0.31* | F (2,12) = 6.210 | 0.0141 |  | 0.60±0.02 | 0.89±0.24 | 0.99±0.20* | F (2,12) = 7.262 | 0.0086 |  | 0.69±0.02 | 1.00±0.27 | 1.12±0.24 | F (2,12) = 6.845 | 0.0104 |
|  | Stria. | 0.24±0.01 | 0.24±0.03 | 0.25±0.05 | F (2,12) = 0.7807 | 0.4942 |  | 0.78±0.03 | 0.88±0.08 | 0.88±0.05 | F (2,12) = 1.111 | 0.381 |  | 0.57±0.02 | 0.63±0.06 | 0.63±0.05 | F (2,12) = 0.6098 | 0.5595 |  | 0.64±0.02 | 0.71±0.06 | 0.71±0.05 | F (2,12) = 1.421 | 0.2793 |
|  |  |  |  |  |  |  |  |  |  |  |  |  |  |  |  |  |  |  |  |  |  |  |  |  |
| **Contralateral** | ***WM:*** |  |  |  |  |  |  |  |  |  |  |  |  |  |  |  |  |  |  |  |  |  |  |  |
|  | CCb | 0.54±0.04 | 0.51±0.04 | 0.51±0.03 | F (2,12) = 0.8951 | 0.4342 |  | 1.19±0.04 | 1.28±0.10 | 1.21±0.04 | F (2,12) = 2.275 | 0.1453 |  | 0.48±0.05 | 0.56±0.07 | 0.53±0.03 | F (2,12) = 3.42 | 0.092 |  | 0.72±0.05 | 0.80±0.07 | 0.76±0.03 | F (2,12) = 3.743 | 0.0785 |
|  | CCs | 0.54±0.02 | 0.46±0.04 | 0.46±0.07 | F (2,12) = 6.216 | 0.0281 |  | 1.11±0.01 | 1.08±0.06 | 1.04±0.07 | F (2,12) = 1.702 | 0.25 |  | 0.45±0.03 | 0.51±0.04 | 0.51±0.04 | F (2,12) = 5.083 | 0.0433 |  | 0.67±0.02 | 0.70±0.04 | 0.69±0.01 | F (2,12) = 2.085 | 0.167 |
|  | ic | 0.61±0.02 | 0.59±0.06 | 0.59±0.05 | F (2,12) = 0.426 | 0.669 |  | 1.25±0.05 | 1.27±0.08 | 1.27±0.09 | F (2,12) =0.1665 | 0.85 |  | 0.42±0.04 | 0.45±0.04 | 0.45±0.02 | F (2,12) = 0.8925 | 0.4352 |  | 0.70±0.04 | 0.72±0.03 | 0.72±0.02 | F (2,12) = 1.044 | 0.4009 |
|  | ab | 0.59±0.05 | 0.50±0.07 | 0.49±0.02 | F(2,12)=  3.996 | 0.0468 |  | 1.16±0.04 | 1.07±0.10 | 1.11±0.05 | F (2,12) = 2.110 | 0.164 |  | 0.47±0.04 | 0.51±0.04 | 0.52±0.01 | F (2,12) = 2.739 | 0.1322 |  | 0.70±0.02 | 0.69±0.04 | 0.72±0.01 | F (2,12) =0.862 | 0.4626 |
|  | ***GM:*** |  |  |  |  |  |  |  |  |  |  |  |  |  |  |  |  |  |  |  |  |  |  |  |
|  | Cortex | 0.20±0.02 | 0.20±0.03 | 0.22±0.03 | F (2,12) = 0.8183 | 0.4793 |  | 0.82±0.03 | 0.89±0.14 | 0.89±0.07 | F (2,12) = 0.9206 | 0.425 |  | 0.61±0.03 | 0.66±0.07 | 0.65±0.03 | F (2,12) = 1.334 | 0.2999 |  | 0.68±0.03 | 0.74±0.09 | 0.73±0.04 | F (2,12) = 1.144 | 0.3509 |
|  | Hippo. | 0.26±0.04 | 0.25±0.03 | 0.27±0.01 | F (2,12) = 1.450 | 0.2973 |  | 0.91±0.07 | 0.99±0.10 | 1.10±0.11 | F (2,12) = 5.083 | 0.043 |  | 0.62±0.02 | 0.67±0.07 | 0.74±0.06 | F (2,12) = 6.647 | 0.0241 |  | 0.71±0.03 | 0.78±0.08 | 0.86±0.08 | F (2,12) = 6.163 | 0.0286 |
|  | Stria. | 0.25±0.02 | 0.26±0.02 | 0.26±0.02 | F (2,12) = 0.08544 | 0.8191 |  | 0.79±0.03 | 0.82±0.03 | 0.82±0.03 | F (2,12) = 0.3977 | 0.6804 |  | 0.56±0.02 | 0.57±0.02 | 0.58±0.01 | F (2,12) =0.0131 | 0.987 |  | 0.64±0.02 | 0.65±0.02 | 0.66±0.01 | F (2,12) =0.172 | 0.8455 |

Abbreviations: CCb, body of the corpus callosum; CCs, splenium of the corpus callosum; ic, internal capsule; ab, anterior commissure; Hippo, hippocampus; Stria.: striatum; WM, white matter; GM, gray matter.

**TABLE S4:** Fractional anisotropy (FA) and axial diffusivity (AD), radial diffusivity (RD), mean diffusivity (MD) from ipsilateral and contralateral hemisphere in sham control (n=6) and rats after mTBI (n=5) or rTBI at **Month 3** (n=6) obtained using *in vivo* DTI.

|  |  | **FA** | | | | |  | **AD (µm^2^/ms)** | | | | |  | **RD (µm^2^/ms)** | | | | |  | **MD (µm^2^/ms)** | | | | |
| --- | --- | --- | --- | --- | --- | --- | --- | --- | --- | --- | --- | --- | --- | --- | --- | --- | --- | --- | --- | --- | --- | --- | --- | --- |
|  |  | **Sham** | **mTBI** | **rTBI** | **F (DFn, DFd)** | **P value** |  | **Sham** | **mTBI** | **rTBI** | **F (DFn, DFd)** | **P value** |  | **Sham** | **mTBI** | **rTBI** | **F (DFn, DFd)** | **P value** |  | **Sham** | **mTBI** | **rTBI** | **F (DFn, DFd)** | **P value** |
| **Ipsilateral** | ***WM:*** |  |  |  |  |  |  |  |  |  |  |  |  |  |  |  |  |  |  |  |  |  |  |  |
|  | CCb | 0.55±0.06 | 0.48±0.11 | 0.44±0.11 | F(2,17)=2.296 | 0.131 |  | 1.22±0.06 | 1.44±0.48 | 1.28±0.14 | F (2,17) = 0.5554 | 0.5906 |  | 0.47±0.05 | 0.71±0.47 | 0.64±0.11* | F (2,17) = 6.574 | 0.015 |  | 0.72±0.03 | 0.95±0.47 | 0.85±0.08 | F (2,17) = 5.736 | 0.0219 |
|  | CCs | 0.56±0.04 | 0.36±0.17 | 0.33±0.06 | F (2,17) = 7.401 | 0.0107 |  | 1.16±0.05 | 1.56±0.47 | 1.16±0.10 | F (2,17) = 4.180 | 0.0334 |  | 0.44±0.04 | 1.00±0.56 | 0.73±0.11 | F (2,17) = 4.054 | 0.0363 |  | 0.68±0.03 | 1.19±0.53 | 0.87±0.10 | F (2,17) = 3.957 | 0.0388 |
|  | ic | 0.65±0.04 | 0.59±0.05 | 0.58±0.05 | F (2,17) = 4.968 | 0.0318 |  | 1.28±0.08 | 1.22±0.08 | 1.19±0.09 | F (2,17) = 2.614 | 0.1221 |  | 0.38±0.04 | 0.44±0.05 | 0.44±0.04 | F (2,17) = 2.933 | 0.0995 |  | 0.68±0.04 | 0.70±0.04 | 0.69±0.04 | F (2,17) = 0.2342 | 0.7954 |
|  | ab | 0.57±0.04 | 0.37±0.12 | 0.36±0.09 | F (2,17) = 9.547 | 0.0048 |  | 1.20±0.17 | 1.50±0.50 | 1.53±0.72 | F (2,17) = 0.7862 | 0.4818 |  | 0.49±0.03 | 0.93±0.51 | 0.94±0.61 | F (2, 17) = 1.961 | 0.1912 |  | 0.73±0.07 | 1.12±0.51 | 1.14±0.65 | F (2,17) = 1.493 | 0.2708 |
|  | ***GM:*** |  |  |  |  |  |  |  |  |  |  |  |  |  |  |  |  |  |  |  |  |  |  |  |
|  | Cortex | 0.19±0.03 | 0.22±0.05 | 0.21±0.01 | F (2,17 ) = 0.9648 | 0.401 |  | 0.82±0.08 | 1.20±0.49 | 1.41±0.79 | F (2,17) = 1.910 | 0.1984 |  | 0.62±0.03 | 0.89±0.39 | 1.05±0.60 | F (2,17) = 1.833 | 0.2097 |  | 0.69±0.05 | 0.99±0.42 | 1.17±0.66 | F (2,17) = 1.869 | 0.2043 |
|  | Hippo. | 0.22±0.02 | 0.22±0.04 | 0.21±0.02 | F (2, 17) = 1.865 | 0.205 |  | 0.88±0.06 | 1.28±0.58 | 1.71±0.29 | F (2,17) = 7.496 | 0.0046 |  | 0.63±0.04 | 0.93±0.47 | 1.27±0.22 | F (2,17) = 7.428 | 0.0048 |  | 0.72±0.04 | 1.04±0.51 | 1.42±0.24 | F (2,17) = 7.522 | 0.0046 |
|  | Stria. | 0.25±0.03 | 0.24±0.03 | 0.24±0.03 | F (2,17) = 1.272 | 0.3206 |  | 0.83±0.06 | 0.85±0.08 | 0.89±0.08 | F (2,17) = 1.052 | 0.3837 |  | 0.59±0.03 | 0.62±0.06 | 0.64±0.05 | F (2,17) = 0.1147 | 0.8928 |  | 0.67±0.04 | 0.70±0.07 | 0.72±0.06 | F (2,17) = 0.06942 | 0.9334 |
|  |  |  |  |  |  |  |  |  |  |  |  |  |  |  |  |  |  |  |  |  |  |  |  |  |
| **Contralateral** | ***WM:*** |  |  |  |  |  |  |  |  |  |  |  |  |  |  |  |  |  |  |  |  |  |  |  |
|  | CCb | 0.57±0.04 | 0.52±0.06 | 0.49±0.05 | F (2,17) = 3.677 | 0.0471 |  | 1.29±0.08 | 1.25±0.08 | 1.21±0.13 | F (2,17) = 1.226 | 0.334 |  | 0.49±0.05 | 0.53±0.06 | 0.54±0.06 | F (2,17) = 2.045 | 0.1801 |  | 0.76±0.05 | 0.77±0.05 | 0.77±0.07 | F (2,17) = 0.1493 | 0.8632 |
|  | CCs | 0.55±0.03 | 0.46±0.07 | 0.41±0.06 | F (2,17) = 10.93 | 0.003 |  | 1.15±0.06 | 1.08±0.07 | 1.03±0.06 | F (2,17) = 5.148 | 0.029 |  | 0.44±0.02 | 0.52±0.06 | 0.55±0.05 | F (2,17) = 12.69 | 0.0018 |  | 0.68±0.02 | 0.71±0.04 | 0.71±0.04 | F (2,17) = 2.260 | 0.1549 |
|  | ic | 0.64±0.03 | 0.58±0.05 | 0.59±0.04 | F (2,17) = 4.108 | 0.035 |  | 1.32±0.08 | 1.26±0.07 | 1.23±0.07 | F (2,17) = 1.928 | 0.176 |  | 0.40±0.03 | 0.46±0.05 | 0.44±0.03 | F (2,17) = 5.097 | 0.0298 |  | 0.71±0.04 | 0.72±0.05 | 0.70±0.03 | F (2,17) = 1.274 | 0.3215 |
|  | ab | 0.57±0.05 | 0.48±0.06 | 0.47±0.05 | F (2, 17) = 6.104 | 0.01 |  | 1.17±0.06 | 1.08±0.09 | 1.09±0.07 | F (2,17) = 3.967 | 0.0539 |  | 0.49±0.06 | 0.53±0.05 | 0.54±0.07 | F (2, 17) = 2.258 | 0.1552 |  | 0.71±0.05 | 0.71±0.04 | 0.72±0.07 | F (2,17) = 0.1137 | 0.8937 |
|  | ***GM:*** |  |  |  |  |  |  |  |  |  |  |  |  |  |  |  |  |  |  |  |  |  |  |  |
|  | Cortex | 0.18±0.02 | 0.21±0.02* | 0.19±0.01 | F (2,17 ) = 4.048 | 0.0365 |  | 0.83±0.03 | 0.87±0.07 | 0.85±0.07 | F (2,17) = 1.076 | 0.3774 |  | 0.64±0.03 | 0.65±0.05 | 0.64±0.05 | F (2,17) = 0.1055 | 0.9009 |  | 0.72±0.06 | 0.72±0.06 | 0.71±0.05 | F (2,17) = 0.3367 | 0.7219 |
|  | Hippo. | 0.24±0.03 | 0.24±0.03 | 0.24±0.03 | F (2, 17) = 0.04337 | 0.9577 |  | 0.90±0.06 | 0.98±0.09 | 1.03±0.14 | F (2,17) = 3.508 | 0.0701 |  | 0.63±0.04 | 0.69±0.07 | 0.72±0.09 | F (2,17) = 3.924 | 0.0552 |  | 0.72±0.04 | 0.79±0.08 | 0.82±0.11 | F (2,17) = 3.951 | 0.0544 |
|  | Stria. | 0.23±0.02 | 0.25±0.03 | 0.25±0.02 | F (2,17) = 1.770 | 0.2197 |  | 0.80±0.06 | 0.83±0.07 | 0.81±0.06 | F (2,17) = 0.1540 | 0.8592 |  | 0.59±0.03 | 0.58±0.03 | 0.58±0.04 | F (2,17) = 1.424 | 0.2857 |  | 0.66±0.04 | 0.66±0.04 | 0.66±0.05 | F (2,17) = 0.2171 | 0.8085 |

Abbreviations: CCb, body of the corpus callosum; CCs, splenium of the corpus callosum; ic, internal capsule; ab, anterior commissure; Hippo, hippocampus; Stria.: striatum; WM, white matter; GM, gray matter.

**TABLE S5:** Fractional anisotropy (FA) and axial diffusivity (AD), radial diffusivity (RD), mean diffusivity (MD) from ipsilateral and contralateral hemisphere in sham control (n=9) and rats after mTBI (n=10) or rTBI at **Month 6** (n=8) obtained using *in vivo* DTI.

|  |  | **FA** | | | | |  | **AD (µm^2^/ms)** | | | | |  | **RD (µm^2^/ms)** | | | | |  | **MD (µm^2^/ms)** | | | | |
| --- | --- | --- | --- | --- | --- | --- | --- | --- | --- | --- | --- | --- | --- | --- | --- | --- | --- | --- | --- | --- | --- | --- | --- | --- |
|  |  | **Sham** | **mTBI** | **rTBI** | **F(DFn,DFd)** | **P value** |  | **Sham** | **mTBI** | **rTBI** | **F (DFn, DFd)** | **P value** |  | **Sham** | **mTBI** | **rTBI** | **F (DFn, DFd)** | **P value** |  | **Sham** | **mTBI** | **rTBI** | **F (DFn, DFd)** | **P value** |
| **Ipsilateral** | ***WM:*** |  |  |  |  |  |  |  |  |  |  |  |  |  |  |  |  |  |  |  |  |  |  |  |
|  | CCb | 0.57±0.02 | 0.42±0.15* | 0.45±0.05* | F (2,24) = 7.411 | 0.0058 |  | 1.23±0.05 | 1.53±0.52 | 1.34±0.18 | F (2,24) = 1.945 | 0.1649 |  | 0.45±0.05 | 0.86±0.59 | 0.66±0.15 | F (2,24) = 2.808 | 0.0802 |  | 0.71±0.05 | 1.08±0.56 | 0.88±0.16 | F (2,24) = 2.547 | 0.0993 |
|  | CCs | 0.54±0.06 | 0.32±0.14* | 0.34±0.08* | F (2,24) = 14.65 | 0.0003 |  | 1.11±0.07 | 1.62±0.60 | 1.29±0.38 | F (2,24) = 4.052 | 0.0392 |  | 0.43±0.05 | 1.10±0.59* | 0.81±0.35 | F (2,24) = 6.964 | 0.0073 |  | 0.66±0.04 | 1.27±0.59* | 0.97±0.36 | F (2,24) = 5.927 | 0.0127 |
|  | ic | 0.66±0.03 | 0.57±0.04* | 0.57±0.06* | F (2,24) = 14.72 | 0.0003 |  | 1.31±0.07 | 1.21±0.06* | 1.23±0.07 | F (2,24) = 6.351 | 0.01 |  | 0.38±0.02 | 0.44±0.04* | 0.46±0.07* | F (2,24) = 9.285 | 0.0024 |  | 0.69±0.03 | 0.70±0.04 | 0.72±0.06 | F (2,24) = 1.268 | 0.3098 |
|  | ab | 0.59±0.04 | 0.41±0.13* | 0.39±0.13* | F (2,24) = 16.43 | 0.0002 |  | 1.21±0.05 | 1.55±0.59 | 1.51±0.65 | F(2,24)=1.991 | 0.171 |  | 0.43±0.05 | 0.90±0.61 | 0.93±0.66 | F (2,24) = 4.455 | 0.0303 |  | 0.69±0.04 | 1.12±0.60 | 1.12±0.66 | F (2, 24) = 3.537 | 0.0552 |
|  | ***GM:*** |  |  |  |  |  |  |  |  |  |  |  |  |  |  |  |  |  |  |  |  |  |  |  |
|  | Cortex | 0.18±0.02 | 0.20±0.05 | 0.21±0.06 | F (2,24) = 0.9411 | 0.4041 |  | 0.80±0.03 | 1.27±0.52 | 1.48±0.69 | F (2,24) = 4.479 | 0.0222 |  | 0.61±0.03 | 0.96±0.42 | 1.11±0.54* | F (2,24) = 3.831 | 0.036 |  | 0.67±0.03 | 1.07±0.45 | 1.23±0.58* | F (2,24) = 4.110 | 0.0448 |
|  | Hippo. | 0.21±0.02 | 0.21±0.04 | 0.21±0.03 | F (2,24) = 2.006 | 0.169 |  | 0.87±0.04 | 1.42±0.57* | 1.67±0.44* | F (2,24) = 10.47 | 0.0014 |  | 0.63±0.03 | 1.05±0.47* | 1.24±0.36* | F (2,24) = 10.03 | 0.0017 |  | 0.71±0.03 | 1.18±0.50* | 1.39±0.39* | F (2,24 = 10.33 | 0.0015 |
|  | Stria. | 0.24±0.02 | 0.24±0.03 | 0.24±0.03 | F (2,24) = 0.8637 | 0.4415 |  | 0.83±0.05 | 0.91±0.15 | 0.93±0.08 | F(2,24)=0.566 | P=-0.5752 |  | 0.59±0.03 | 0.66±0.11 | 0.66±0.06 | F (2,24) = 0.5667 | 0.5791 |  | 0.67±0.04 | 0.74±0.12 | 0.75±0.06 | F (2,24) = 0.5525 | 0.5826 |
|  |  |  |  |  |  |  |  |  |  |  |  |  |  |  |  |  |  |  |  |  |  |  |  |  |
| **Contralateral** | ***WM:*** |  |  |  |  |  |  |  |  |  |  |  |  |  |  |  |  |  |  |  |  |  |  |  |
|  | CCb | 0.56±0.04 | 0.49±0.06 | 0.51±0.03 | F (2,24) = 5.414 | 0.017 |  | 1.28±0.09 | 1.28±0.17 | 1.26±0.07 | F (2,24) = 0.06417 | 0.938 |  | 0.49±0.05 | 0.57±0.10 | 0.55±0.03 | F (2,24) = 3.364 | 0.0516 |  | 0.75±0.06 | 0.81±0.12 | 0.79±0.04 | F (2,24) = 1.195 | 0.3202 |
|  | CCs | 0.57±0.03 | 0.43±0.06 | 0.41±0.04 | F (2,24) = 36.06 | <0.0001 |  | 1.16±0.05 | 1.06±0.06 | 1.02±0.04 | F (2,24) = 16.45 | <0.0001 |  | 0.42±0.04 | 0.54±0.06 | 0.53±0.04 | F (2,24) = 19.75 | <0.0001 |  | 0.67±0.03 | 0.72±0.05 | 0.69±0.03 | F (2,24) = 3.551 | 0.0546 |
|  | ic | 0.60±0.04 | 0.60±0.04 | 0.58±0.05 | F (2,24) = 0.9290 | 0.4165 |  | 1.27±0.08 | 1.30±0.10 | 1.27±0.09 | F (2,24) = 0.2933 | 0.7484 |  | 0.43±0.03 | 0.45±0.03 | 0.46±0.08 | F (2,24) = 1.516 | 0.2514 |  | 0.71±0.03 | 0.73±0.04 | 0.73±0.07 | F (2,24) = 0.6995 | 0.5123 |
|  | ab | 0.56±0.03 | 0.49±0.08 | 0.50±0.04 | F (2,24) = 4.238 | 0.0348 |  | 1.19±0.06 | 1.13±0.12 | 1.13±0.07 | F(2,24)=1.166 | 0.3384 |  | 0.46±0.04 | 0.53±0.07 | 0.51±0.06 | F (2,24) = 4.494 | 0.0296 |  | 0.70±0.03 | 0.73±0.05 | 0.71±0.06 | F (2, 24) = 0.9817 | 0.3892 |
|  | ***GM:*** |  |  |  |  |  |  |  |  |  |  |  |  |  |  |  |  |  |  |  |  |  |  |  |
|  | Cortex | 0.18±0.02 | 0.17±0.02 | 0.17±0.03 | F (2,24) = 0.6577 | 0.5271 |  | 0.83±0.05 | 0.87±0.07 | 0.84±0.04 | F (2,24) = 1.360 | 0.2758 |  | 0.64±0.04 | 0.67±0.05 | 0.65±0.03 | F (2, 24) = 2.518 | 0.1141 |  | 0.70±0.04 | 0.74±0.05 | 0.72±0.03 | F (2,24) = 2.066 | 0.1612 |
|  | Hippo. | 0.23±0.02 | 0.24±0.04 | 0.24±0.03 | F (2,24) = 0.7573 | 0.4861 |  | 0.91±0.05 | 1.01±0.10 | 1.09±0.11 | F (2,24) = 6.778 | 0.0046 |  | 0.64±0.04 | 0.70±0.06 | 0.75±0.09 | F (2, 24) = 5.404 | 0.0115 |  | 0.73±0.04 | 0.81±0.07 | 0.87±0.09 | F (2,24) = 6.249 | 0.0065 |
|  | Stria. | 0.24±0.02 | 0.23±0.02 | 0.24±0.03 | F (2,24) = 0.8616 | 0.4351 |  | 0.82±0.05 | 0.84±0.06 | 0.83±0.02 | F (2,24) = 0.7403 | 0.4936 |  | 0.58±0.03 | 0.60±0.02 | 0.59±0.03 | F (2,24) = 1.632 | 0.2284 |  | 0.66±0.03 | 0.68±0.03 | 0.67±0.02 | F (2,24) = 1.269 | 0.3096 |

Abbreviations: CCb, body of the corpus callosum; CCs, splenium of the corpus callosum; ic, internal capsule; ab, anterior commissure; Hippo, hippocampus; Stria.: striatum; WM, white matter; GM, gray matter.

**TABLE S6:** The correlation between contusion (or lesion) volume (by T2WI) and fractional anisotropy (FA), radial diffusivity (RD), and mean diffusivity (MD) in splenium of the corpus callosum (CCs) and anterior commissure (ab) in both TBI, and rmTBI group rats obtained during day 3 to month 9 of after surgery.

| DTI parameters & regions  Time & correlation | | FA | | RD | | MD | |
| --- | --- | --- | --- | --- | --- | --- | --- |
|  |  | CCs | ab | CCs | ab | CCs | ab |
| D3 | P | 0.6112 | 0.8584 | 0.9476 | 0.9366 | 0.7146 | 0.6574 |
|  | R^2^ | 0.04571 | 0.005749 | 0.000783 | 0.001145 | 0.02392 | 0.03499 |
| M1 | P | 0.0022* | 0.0038* | 0.0122* | 0.0239* | 0.0315* | 0.0389* |
|  | R^2^ | 0.5261 | 0.4873 | 0.3941 | 0.3346 | 0.3087 | 0.2886 |
| M3 | P | <0.0001* | <0.0001* | 0.0003* | 0.0011* | 0.0017* | 0.0028* |
|  | R^2^ | 0.6769 | 0.5968 | 0.5329 | 0.4528 | 0.4287 | 0.3984 |
| M6 | P | <0.0001* | <0.0001* | <0.0001* | 0.0002* | <0.0001* | 0.0004* |
|  | R^2^ | 0.7457 | 0.5799 | 0.6695 | 0.4259 | 0.5868 | 0.3948 |
| M9 | P | <0.0001* | <0.0001* | <0.0001* | <0.0001* | <0.0001* | <0.0001* |
|  | R^2^ | 0.7278 | 0.7281 | 0.6605 | 0.5577 | 0.5777 | 0.5091 |

**P* significant <0.05. The *R-squared value, denoted by R^2^*, is the square of the correlation.
